# Understanding the Photophysical Properties of the Polyoxotitanates Ln_2_Ti_4_O_6_(phen)_2_(met)_10_

**DOI:** 10.1021/acs.inorgchem.5c02216

**Published:** 2025-07-31

**Authors:** Rosa Müller, Alasdair Tew, Andrew D. Bond, Akshay Rao, Hugo Bronstein, Dominic S. Wright

**Affiliations:** † Yusuf Hamied Department of Chemistry, 2152 Cambridge University, Cambridge CB2 1EW, U.K.; ‡ The Cavendish Laboratory, Department of Physics, Cambridge University, Cambridge CB3 0HE, U.K.

## Abstract

Understanding and tuning the luminescent properties of lanthanide­(III) ions is of great interest for a range of optical applications, for example photo down-shifters for photovoltaics. In this work a series of isostructural polyoxotitanates (POTs) of general formula Ln_2_Ti_4_O_6_(phen)_2_(met)_10_ (**Ln**_**2**_ **Ti**_**4**_, Ln = Nd, Sm–Er, Yb, met = methacrylate) was synthesized, where the lanthanide is coordinated to a titanium-oxo core and the antenna ligand 1,10-phenanthroline (phen). The energies of the ligand S_1_ and T_1_ states in the **Ln**_**2**_ **Ti**_**4**_ series were determined, which, together with the luminescence spectra of the **Ln**_**2**_ **Ti**_**4**_ compounds, gave new insights into the intramolecular energy-transfer mechanism and efficiencies between the ligand periphery and the Ln^3+^ ions. The effect of the rigidity of the Ti_4_O_6_-core on the coordination environment of a lanthanide was quantified for the first time using lifetime measurements, which shows a significant increase of Δτ = 1.1 ms for **Eu**_**2**_ **Ti**_**4**_ compared to the reference Eu_2_(phen)_2_(met)_6_ (**Eu**_**2**_), in which no metal-oxo core is present. This key finding illustrates the advantage in designing lanthanide-based lumiphores containing rigid metal-oxide cores.

## Graphical abstract

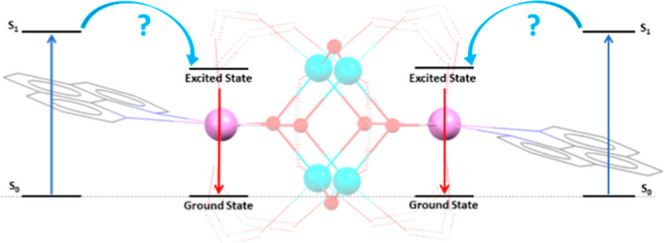

## Introduction

The unique optical properties of the lanthanides have made them indispensable in the modern world for a wide range of light-management applications such as telecommunications, lighting and bioimaging.^1^ Despite having very similar physical properties due to the core-like character of the 4f-electrons and their similar atomic/ionic sizes, the different electronic configurations of the elements give rise to a variety of luminescence and magnetic behaviors.^2^ Elements such as europium and terbium emit in the visible region of the electromagnetic spectrum and are therefore of fundamental use for LEDs and as anticounterfeit materials.^3^ In a similar way near-infrared (NIR)-emitters (e.g., neodymium, erbium, ytterbium) find applications in bioimaging techniques, lasers and communications.^4^ Dysprosium and holmium on the other hand make frequent appearances in single-molecule magnets due to their large total angular momentum, *J*.^5^

A major hurdle to overcome in the field of lanthanide emission is the inefficiency of the symmetry-forbidden 4f–4f transitions which leads to low molar absorption coefficients and therefore low emission intensities. This problem can be solved by indirectly exciting the lanthanide using an intramolecular energy transfer process from an organic antenna ligand directly bonded to lanthanide ions which sidesteps the Laporte rule.^6^ This so-called ‘antenna effect’ has been studied in great detail, e.g., for down-shifting europium complexes which have been proposed as additives to plastic sheeting in polytunnels to accelerate plant growth in an agricultural setting, by transferring unused UV light to photosynthetically active red light.^7^ The position of the antenna ligand S_1_ and T_1_ states relative to the excited f-states of the complexed lanthanide ion is critical to guarantee efficient sensitization (Figure 1).^8^ Equally important is minimizing nonradiative decay processes via multiphonon relaxation, which in general means avoiding highly oscillating OH, NH, and CH bonds in the proximity of the lanthanide center.^6b^ The overall quantum yield Φ_Ln_^L^ of the luminescence process is given as

1 $$\Phi_{\mathrm{Ln}}^{L}=\eta_{sens}\cdot \Phi_{\mathrm{Ln}}^{\mathrm{Ln}}=\eta_{sens}\cdot \frac{\tau_{\exp}}{\tau_{rad}}$$

**Figure 1:**
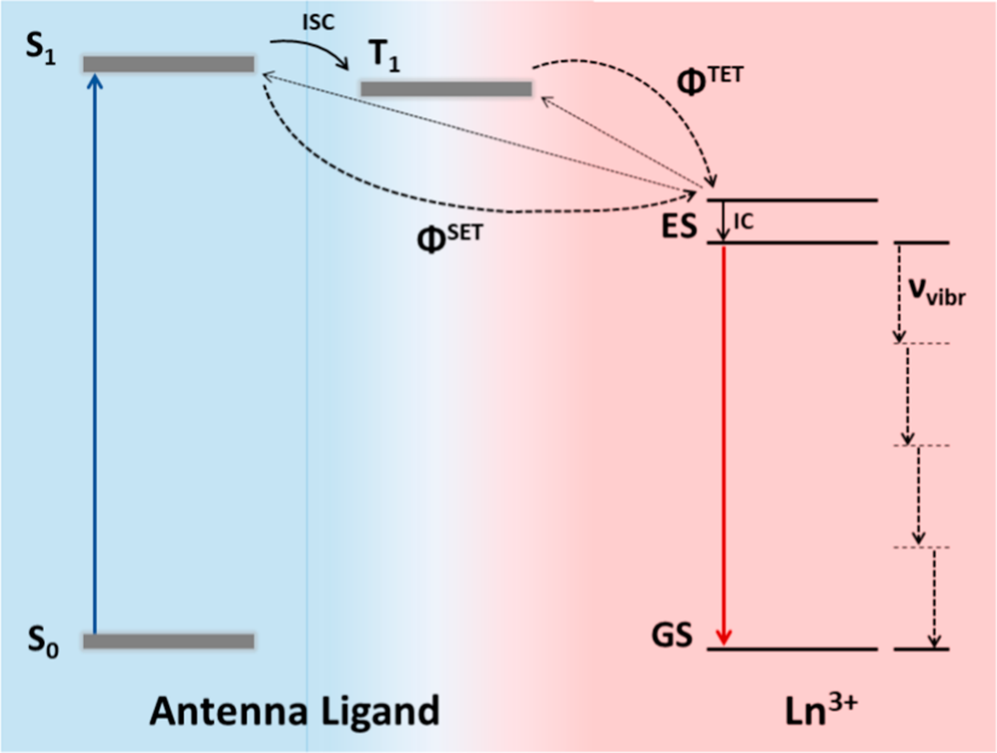
Schematic diagram of the intramolecular energy-transfer (ET) processes between an antenna ligand and a lanthanide (III) ion. In the first step the ligand absorbs a high-energy photon into its S_1_ state, from which either direct or indirect sensitization of the lanthanide can occur (via intersystem crossing, ISC, to an intermediate antenna triplet state T_1_).^9^ The efficiency of these intramolecular ET processes is described by η_sens_ = Φ^SET^ + Φ^ISC^·Φ^TET^ (SET = singlet ET and TET = triplet ET). In the case of insufficient energy separation of the states involved in the ET (<10*k*_B_ *T* ≈ 2000 cm^–1^) a dynamic back-transfer to the ligand becomes possible.^8^ Upon population of one or multiple excited state(s) (ES) and subsequent internal conversion (IC) to the lowest ES, the Ln^3+^ can then emit a photon which is red-shifted compared to the ligand absorption to reach the ground state (GS). An alternative relaxation pathway is the nonradiative multiphonon relaxation shown to the right of the figure where the excited electron resonates with the surrounding ligand OH-, NH- or CH-bond vibration frequencies (ν_vibr_).

Where η_sens_ is the total sensitization efficiency and Φ_Ln_^Ln^ the intrinsic quantum yield of the lanthanide.^6b^

Our current interest is in the potential use of lanthanide-based luminescence in solar cells, and in this work, we focus on the lanthanide-dependent efficiency of the intramolecular energy transfer processes between a well-known photosensitizer (1,10-phenanthroline) and a coordinated Ln^3+^ ion. Rather than studying traditional coordination compounds we investigate the impact of coordinating lanthanide ions to a polyoxotitanate (POT) framework, having a rigid core structure, which should minimize nonradiative vibrations and therefore enhance luminescence by increasing the radiative lifetime. Long-lifetime compounds are pivotal to, e.g., the detection sensitivity in bioimaging techniques where samples often have a highly fluorescent background.^1a,10^

Based on the synthetic method used to obtain the previously reported POT [Eu_2_Ti_4_O_6_(phen)_2_(met)_10_][Bibr ref11] we synthesized a series of isostructural cages [Ln_2_Ti_4_O_6_(phen)_2_(met)_10_] [**Ln**_**2**_ **Ti**_**4**_: Ln = Nd (**Nd**_**2**_ **Ti**_**4**_), Sm (**Sm**_**2**_ **Ti**_**4**_), Eu (**Eu**_**2**_ **Ti**_**4**_), Gd (**Gd**_**2**_ **Ti**_**4**_), Tb (**Tb**_**2**_ **Ti**_**4**_), Dy (**Dy**_**2**_ **Ti**_**4**_), Ho (**Ho**_**2**_ **Ti**_**4**_), Er (**Er**_**2**_ **Ti**_**4**_), Yb (**Yb**_**2**_ **Ti**_**4**_)] and analyzed their structural and luminescence behavior. We picked this system due to its synthetic accessibility and chose not to alter the auxiliary ligands (methacrylate) as these are not expected to contribute to lanthanide sensitization. In addition to this series of cages, two additional related structural types with varying sizes of the titanium-oxo core were also isolated as minor components in the **Ln**_**2**_ **Ti**_**4**_ cages, depending on the reaction conditions (stoichiometry and moisture). Despite successful sensitization of all lanthanides via the phenanthroline antenna ligand the efficiency of the luminescence processes in the **Ln**_**2**_ **Ti**_**4**_ cages varies significantly depending on the electronic structure of the respective Ln^3+^ ion present. The lifetime of the excited state in Eu^3+^ was significantly increased upon encapsulating the Ln-phen unit into a POT framework (compared to without the metal-oxo core), confirming the effect of the rigidity of the POT core on reducing nonradiative (vibronic) decay pathways. Further important insights into the intramolecular energy transfer mechanism between the ligand and the lanthanide in the **Ln**_**2**_ **Ti**_**4**_ cages were also gained, although these studies were unable to confirm the involvement of a ligand triplet state. Overall, this work demonstrates the positive impact of a rigid molecular environment on the emissive properties of Ln^3+^ ions as well as providing important information on their phenanthroline-mediated sensitization.

## Experimental Section

### General Remarks

All experimental synthesis procedures were carried out under a dry inert atmosphere of N_2_ on a vacuum-line and glovebox (Saffron, type α) unless specified otherwise. Starting materials were purchased from suppliers (Fischer Scientific, Alpha Aesar, Merck) and used as received. Elemental analysis was performed using a PerkinElmer 240 Elemental Analyzer. IR spectra were acquired on a Thermo Scientific Nicolet iS50 FT-IR in ATR mode. Powder XRD data were collected on a Malvern Panalytical Empyrean diffractometer or a Bruker D8 Advance diffractometer (both Cu radiation). High-resolution powder XRD data were collected at the Diamond Light Source Synchrotron i11 beamline using a wavelength of 0.82443 Å.

### Synthesis of Compounds

#### General Procedure for Ln_2_Ti_4_

The synthesis of the novel compounds was carried out following a procedure described in the literature with slight modifications.^11^ Ln­(OAc)_3_·*x*H_2_O (0.06 mmol, 1 equiv) and 1,10-phenanthroline (120 mg, 0.67 mmol, 11 equiv) were added to the Teflon liner (12 mL) of a steel autoclave and dissolved in anhydrous MeCN (2.5 mL). After the addition of Ti­(O^*i*^Pr)_4_ (0.35 mL, 1.18 mmol, 20 equiv) and methacrylic acid (0.5 mL, 5.89 mmol, excess), the autoclave was sealed and heated to 80 °C for 5 days. A clear yellow solution was obtained and left under ambient conditions for slow evaporation over 1–2 weeks to give the compounds as colorless block-shaped crystals. The product was washed thoroughly with MeCN to remove unreacted starting material and side products and dried under vacuum.

##### [Nd_2_Ti_4_O_6_(phen)_2_(met)_10_]

Colorless crystals (71% yield based on Nd). Anal. Calcd for Nd_2_Ti_4_C_64_O_26_H_66_N_4_: C, 43.0; H, 3.7; N, 3.1. Found: C, 42.4; H, 3.6; N, 3.0.

##### [Sm_2_Ti_4_O_6_(phen)_2_(met)_10_]

Colorless crystals (46% yield based on Sm). Anal. Calcd for Sm_2_Ti_4_C_64_O_26_H_66_N_4_: C, 42.7; H, 3.7; N, 3.1. Found: C, 42.5; H, 3.7; N, 3.1.

##### [Eu_2_Ti_4_O_6_(phen)_2_(met)_10_]

Colorless crystals (74% yield based on Eu). Anal. Calcd for Eu_2_Ti_4_C_64_O_26_H_66_N_4_: C, 42.6; H, 3.7; N, 3.1. Found: C, 42.3; H, 3.6; N, 3.5.

##### [Gd_2_Ti_4_O_6_(phen)_2_(met)_10_]

Colorless crystals (42% yield based on Gd). Anal. Calcd for Gd_2_Ti_4_C_64_O_26_H_66_N_4_: C, 42.4; H, 3.7; N, 3.1. Found: C, 41.8; H, 3.5; N, 3.0.

##### [Tb_2_Ti_4_O_6_(phen)_2_(met)_10_]

Colorless crystals (40% yield based on Tb). Anal. Calcd for Tb_2_Ti_4_C_64_O_26_H_66_N_4_: C, 42.3; H, 3.7; N, 3.1. Found: C, 42.3; H, 3.7; N, 3.2.

##### [Dy_2_Ti_4_O_6_(phen)_2_(met)_10_]

Colorless crystals (36% yield based on Dy). Anal. Calcd for Dy_2_Ti_4_C_64_O_26_H_66_N_4_: C, 42.2; H, 3.7; N, 3.1. Found: C, 42.1; H, 3.8; N, 2.6.

##### [Ho_2_Ti_4_O_6_(phen)_2_(met)_10_]

Pale yellow crystals (72% yield based on Ho). Anal. Calcd for Ho_2_Ti_4_C_64_O_26_H_66_N_4_: C, 42.0; H, 3.6; N, 3.1. Found: C, 42.1; H, 3.6; N, 3.0.

##### [Er_2_Ti_4_O_6_(phen)_2_(met)_10_]

Pale pink crystals (49% yield based on Er). Anal. Calcd for Er_2_Ti_4_C_64_O_26_H_66_N_4_: C, 41.9; H, 3.6; N, 3.1. Found: C, 42.1; H, 3.7; N, 3.0.

##### [Yb_2_Ti_4_O_6_(phen)_2_(met)_10_]

Colorless crystals (86% yield based on Yb). Anal. Calcd for Yb_2_Ti_4_C_64_O_26_H_66_N_4_: C, 41.7; H, 3.6; N, 3.0. Found: C, 40.9; H, 3.7; N, 2.5.

#### General Procedure for Ln_2_

Ln­(OAc)_3_·*x*H_2_O (0.04 mmol, 1 equiv) and 1,10-phenanthroline (6.5 mg, 0.04 mmol, 1 equiv) were added to the Teflon liner (12 mL) of a steel autoclave and dissolved in anhydrous MeCN (1.0 mL). After the addition of methacrylic acid (0.02 mL, 0.22 mmol, 6 equiv) the autoclave was sealed and heated to 80 °C for 5 days. The obtained clear yellow solution was left under ambient conditions for slow evaporation over 4–5 days to give the compounds as colorless needles, which were washed with MeCN and dried under vacuum.

##### [Eu_2_(phen)_2_(met)_6_(Hmet)_2_]

Colorless crystals (48% yield based on Eu). Anal. Calcd for Eu_2_C_56_O_16_H_58_N_4_: C, 49.9; H, 4.3; N, 4.2. Found: C, 49.6; H, 4.2; N, 3.5.

##### [Ho_2_(phen)_2_(met)_6_(Hmet)_2_]

Colorless crystals (42% yield based on Ho). Anal. Calcd for Ho_2_C_56_O_16_H_58_N_4_: C, 49.0; H, 4.3; N, 4.1. Found: C, 49.0; H, 4.2; N, 4.1.

##### [Yb_2_(phen)_2_(met)_6_(Hmet)_2_]

Colorless crystals (36% yield based on Yb). Anal. Calcd for Yb_2_C_48_O_12_H_46_N_4_: C, 47.4; H, 3.8; N, 4.6. Found: C, 47.5; H, 3.7; N, 4.6.

##### [Sm_2_(phen)_2_(met)_6_(Hmet)_2_], [Gd_2_(phen)_2_(met)_6_(Hmet)_2_], [Er_2_(phen)_2_(met)_6_(Hmet)_2_]

These compounds were analyzed via pXRD, but no further bulk analysis was carried out.

#### X-ray Crystallographic Study

Single-crystal X-ray data were collected on a Bruker D8-QUEST diffractometer, equipped with an Incoatec IμS Cu microsource (λ = 1.5418 Å) and a PHOTON-III detector operating in shutterless mode. Crystals were mounted on a MiTeGen crystal mount using inert polyfluoroether oil and the analysis was carried out under an Oxford Cryosystems open-flow N_2_ Cryostream. Structures were solved using SHELXT and refined using SHELXL.^12,13^ Details of the data collections and structural refinements are given in Tables S1 and S5 in the Supporting Information. The crystal structures have been deposited with the Cambridge Crystallographic Data Centre (CCDC 2430472–2430485).

#### Absorption and Emission Measurements

Absorption measurements were acquired using a Shimadzu UV-3600 Plus in 10 mm quartz cuvettes. Samples were dissolved in DCM under ambient atmosphere. Fluorescence measurements were carried out using an Edinburgh Instruments FLS1000 with a xenon lamp as excitation source. Lanthanide lifetimes were measured on the same setup using a monochromated microsecond flashlamp as the excitation source. Both Si and InGaAs photomultiplier tubes were employed as the detectors for the visible and infrared, respectively.

#### Transient Absorption Measurements

TA measurements utilized a PHAROS (Light Conversion) 1030 nm femtosecond laser as the fundamental beam. The pump pulse was generated in an ORPHEUS (Light Conversion) OPA at a wavelength of 320 nm. The delay, white light generation and detection were performed in a HARPIA (light conversion) TA system.

## Results and Discussion

### Synthesis and Crystallography

The new cages of general formula [Ln_2_Ti_4_O_6_(phen)_2_(met)_10_] [**Ln**_**2**_ **Ti**_**4**_; Ln = Nd (**Nd**_**2**_ **Ti**_**4**_), Sm (**Sm**_**2**_ **Ti**_**4**_), Gd (**Gd**_**2**_ **Ti**_**4**_), Tb (**Tb**_**2**_ **Ti**_**4**_), Dy (**Dy**_**2**_ **Ti**_**4**_), Ho (**Ho**_**2**_ **Ti**_**4**_), Er (**Er**_**2**_ **Ti**_**4**_), Yb (**Yb**_**2**_ **Ti**_**4**_)] and the previously reported Eu cage for Ln = Eu (**Eu**_**2**_ **Ti**_**4**_) were synthesized under inert-atmosphere solvothermal conditions in an autoclave by the reaction of Ti­(O^*i*^Pr)_4_ (20 equiv) with Ln­(OAc)_3_·*x*H_2_O (1 equiv), 1,10-phenanthroline (11 equiv) and methacrylic acid (excess) in anhydrous acetonitrile following the conditions reported for **Eu**_**2**_ **Ti**_**4**_.^11^ They were characterized using elemental analysis, IR spectroscopy, powder (pXRD) and single-crystal (scXRD) diffraction prior to detailed photophysical investigations (Figures S1–S5). The block-shaped crystals obtained from the reactions were stable under ambient conditions and moderately soluble in aprotic organic solvents (dichloromethane). ScXRD analysis of **Nd**_**2**_ **Ti**_**4**_, **Sm**_**2**_ **Ti**_**4**_, **Eu**_**2**_ **Ti**_**4**_, **Gd**_**2**_ **Ti**_**4**_, **Dy**_**2**_ **Ti**_**4**_, **Ho**_**2**_ **Ti**_**4**_, **Er**_**2**_ **Ti**_**4**_, and **Yb**_**2**_ **Ti**_**4**_ showed that they are isostructural (Figure 2a) and their bulk purity was confirmed via pXRD (Figures S3 and S4).

**Figure 2:**
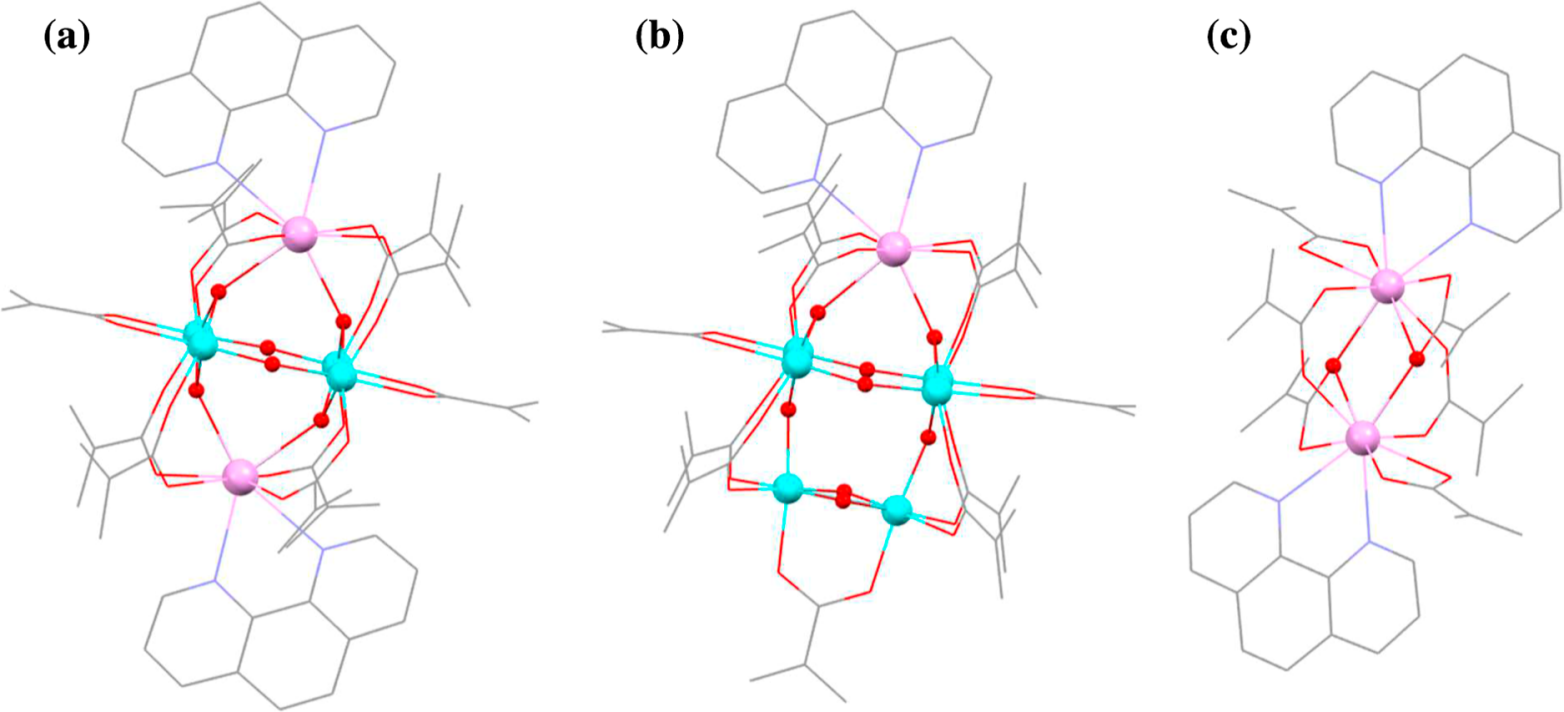
The three structural types obtained from the reactions of Ti­(O^*i*^Pr)_4_ with Ln­(OAc)_3_·*x*H_2_O, 1,10-phenanthroline and methacrylic acid under varying reaction conditions. Single-crystal structures of (a) the isostructural cages [Ln_2_Ti_4_O_6_(phen)_2_(met)_10_] [**Ln**_**2**_ **Ti**_**4**_, Ln = Nd, Sm, Eu, Gd, Tb, Dy, Ho, Er, Yb; the structure shown is that of **Nd**_**2**_ **Ti**_**4**_], (b) [LnTi_6_O_8_(phen)­(met)_11_] (**LnTi**_**6**_, Ln = Sm, Gd, Tb, Ho, Er; the structure shown is that of **SmTi**_**6**_), and (c) [Ln_2_(phen)_2_(met)_6_] (**Ln**_**2**_, Ln = Eu, Ho, Yb; data shown for Ln = Eu). Both **Ln**_**2**_ **Ti**_**4**_ and **LnTi**_**6**_ contain Ti­(IV) only. H atoms are omitted for clarity. A consistent coloring scheme is used throughout this work: Ti = light blue, Ln = pink, O = red, C = gray, N = dark blue.

Further examination of the bulk samples of the **Ln**_**2**_ **Ti**_**4**_ cages using scXRD showed that the new cage compounds [LnTi_6_O_8_(phen)­(met)_11_] [**LnTi**_**6**_, Ln = Sm (**SmTi**_**6**_), Gd (**GdTi**_**6**_), Ho (**HoTi**_**6**_), Er (**ErTi**_**6**_)] are present as minor components in the corresponding **Ln**_**2**_ **Ti**_**4**_ reactions, distinguishable from the **Ln**_**2**_ **Ti**_**4**_ cages by their different crystal morphologies (**LnTi**_**6**_, indistinct globules; **Ln**_**2**_ **Ti**_**4**_, clear blocks) (Figure 2b). Repeated reactions under strictly inert conditions showed that the **LnTi**_**6**_ cages are present in only trace amounts in bulk samples of **Sm**_**2**_ **Ti**_**4**_, **Gd**_**2**_ **Ti**_**4**_, **Ho**_**2**_ **Ti**_**4**_, and **Er**_**2**_ **Ti**_**4**_, as shown by pXRD analysis. For Ln = Tb, more variable behavior was observed. pXRD indicated that [TbTi_6_O_8_(phen)­(met)_11_] (**TbTi**_**6**_) was generally the major product, while elemental analysis (C, H, N) more closely matched that predicted for **Tb**_**2**_ **Ti**_**4**_ which we were able to isolate as single-crystals from these samples. The reason for the variability appears to stem from the variable presence of adventitious water, present in the reaction solvent and as water of hydration in the Ln­(OAc)_3_.*x*H_2_O precursors employed. It can be noted in this regard that Tb­(OAc)_3_·*x*H_2_O appears to be particularly hygroscopic. The different cages obtained in these reactions are summarized in Scheme 1. It does *not* appear that the balance between **Ln**_**2**_ **Ti**_**4**_ and **LnTi**_**6**_ is Ln-dependent (e.g., on ionic size).

**Scheme 1:**
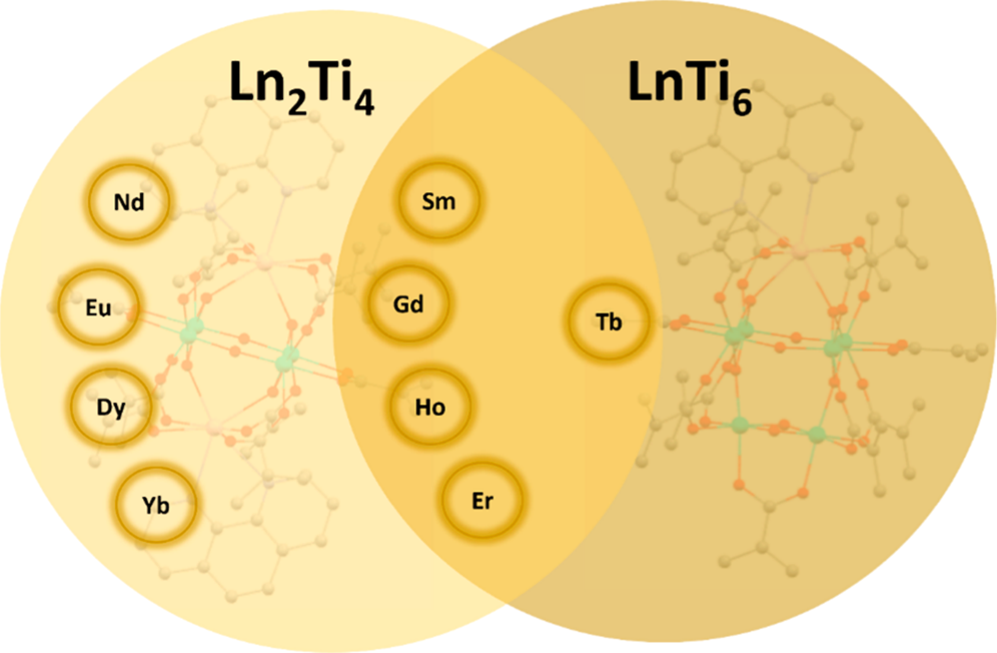
An Overview Showing the Structural Types Found for Each Lanthanide in the Reaction of Ti­(O^*i*^Pr)_4_, Ln­(OAc)_3_·*x*H_2_O, 1,10-Phenanthroline and Methacrylic Acid^a^

a For Ln = Nd, Eu, Dy and Yb the **Ln**_**2**_ **Ti**_**4**_ cage was found exclusively in both sc- and pXRD. In the case of Ln = Sm, Gd, Ho and Er both **Ln**_**2**_ **Ti**_**4**_ and **LnTi**_**6**_ cages were observed in scXRD, but bulk analysis shows the **Ln**_**2**_ **Ti**_**4**_ compounds to be the major component. In the case of Ln = Tb both structural types were found in scXRD, but the **LnTi**_**6**_ cage appeared to be the main reaction product.

In further studies, we looked at the potential influence of reaction stoichiometry on the product(s) formed in these reactions. Increasing the amount of Ti/Ln in all of these reactions does not change the quantity of **LnTi**_**6**_ cages formed, even when using a large excess of Ti­(O^*i*^Pr)_4_. While reducing the amount of Ti/Ln to 2:1 led to the formation of the titanium-free compounds [Ln_2_(phen)_2_(met)_6_] (**Ln**_**2**_, Ln = Sm, Eu, Gd, Ho, Er, Yb) independently of the size of the lanthanide (Figure 2c).

The Ln_2_Ti_4_O_6_-core structure of the **Ln**_**2**_ **Ti**_**4**_ cages is well documented and various cages possessing the same arrangement and stoichiometry have been reported for Ln = Eu.^14^ Analysis of the compound geometry along the series of lanthanides in our case shows no significant change of the metal-oxo core or the Ln–N (phenanthroline) bond lengths. However, the orientation of the coligands, in this case methacrylate, tends to vary slightly between the compounds, especially when aromatic carboxylates such as 4-*tert*-butylbenzoate are used.^14c^ The **LnTi**_**6**_-type POTs are structurally related to the **Ln**_**2**_ **Ti**_**4**_-type compounds: the ‘top’ fragment [Ln­(phen)­(μ_3_-O)_2_Ti_2_(μ_2_-O)_2_(met)_6_] is identical in both **Ln**_**2**_ **Ti**_**4**_ and **LnTi**_**6**_. The second Ln­(phen) unit present in the **Ln**_**2**_ **Ti**_**4**_ series is replaced by a Ti_2_(μ_2_-O)_2_(met) fragment in the **LnTi**_**6**_ cage structure. The **LnTi**_**6**_ cage is mirror-symmetric, with the phen ligand lying in the mirror plane. Single-crystals of the dimeric Ln_2_(phen)_2_(met)_6_ (**Ln**_**2**_) structural type were analyzed for Ln = Eu, Ho and Yb. All three structurally characterized **Ln**_**2**_ cages have four Ln–Ln bridging methacrylate ligands and one terminal 1κ^2^O-methacrylate ligand per lanthanide. For Ln = Eu and Ho two of the four bridging ligands have a 1:2κ^2^O; 1κ^2^OC­(C_3_H_5_) coordination mode whereas for Ln = Yb each methacrylate coordinates as (1κO, 2κO) (Figure 2c). This variation in coordination mode of the carboxylate ligands is also seen in previously reported compounds such as [La_2_(phen)_2_(met)_6_], [Ln_2_(phen)_2_(O_2_CCCl_3_)_6_(HOEt)_2_] (Ln = Nd, Pr, Er) and [Nd_2_(TFSA)_3_(phen)_2_]_*n*_ (TFSA = tetrafluorosuccinate).^15^ In some cases, even compounds with two bridging and four terminal carboxylate ligands have been obtained.^16^

### Optical Properties

Samples for photophysical measurements were analyzed by pXRD and elemental analysis to confirm phase purity prior to analysis. However, in the case of **Tb**_**2**_ **Ti**_**4**_ there is some uncertainty as to the exact level of contamination with **TbTi**_**6**_ due to its formation in variable amounts (as noted above). To start with, the absorption and emission spectra of all novel POT compounds (**Ln**_**2**_ **Ti**_**4**_ with Ln = Nd, Sm, Eu, Gd, Tb, Dy, Ho, Er, Yb) and **Eu**_**2**_ were recorded (Figure 3). The excitation spectra for the lanthanide-centered emission were measured for a few selected compounds and showed good overlap with the ligand-based absorption (Figure S6). The samples showed signs of decomposition upon exposure to the laser beam within a few minutes after their preparation (Figures S7 and S8b). The observed spectral changes in **Eu**_**2**_ and **Ln**_**2**_ **Ti**_**4**_ were similar in the absorption spectrum (loss of the shoulder around 290 nm) but varied in the emission. For **Ln**_**2**_ **Ti**_**4**_ the residual phenanthroline emission decreases gradually over time, whereas for **Eu**_**2**_ the acene-like emission between 340 and 420 nm increases, which suggests different degradation pathways. It should also be noted that we did not observe any changes in the ^1^H NMR spectra for both types of compounds, which might indicate that the decomposition during the photophysical measurements is radiation-induced.

**Figure 3:**
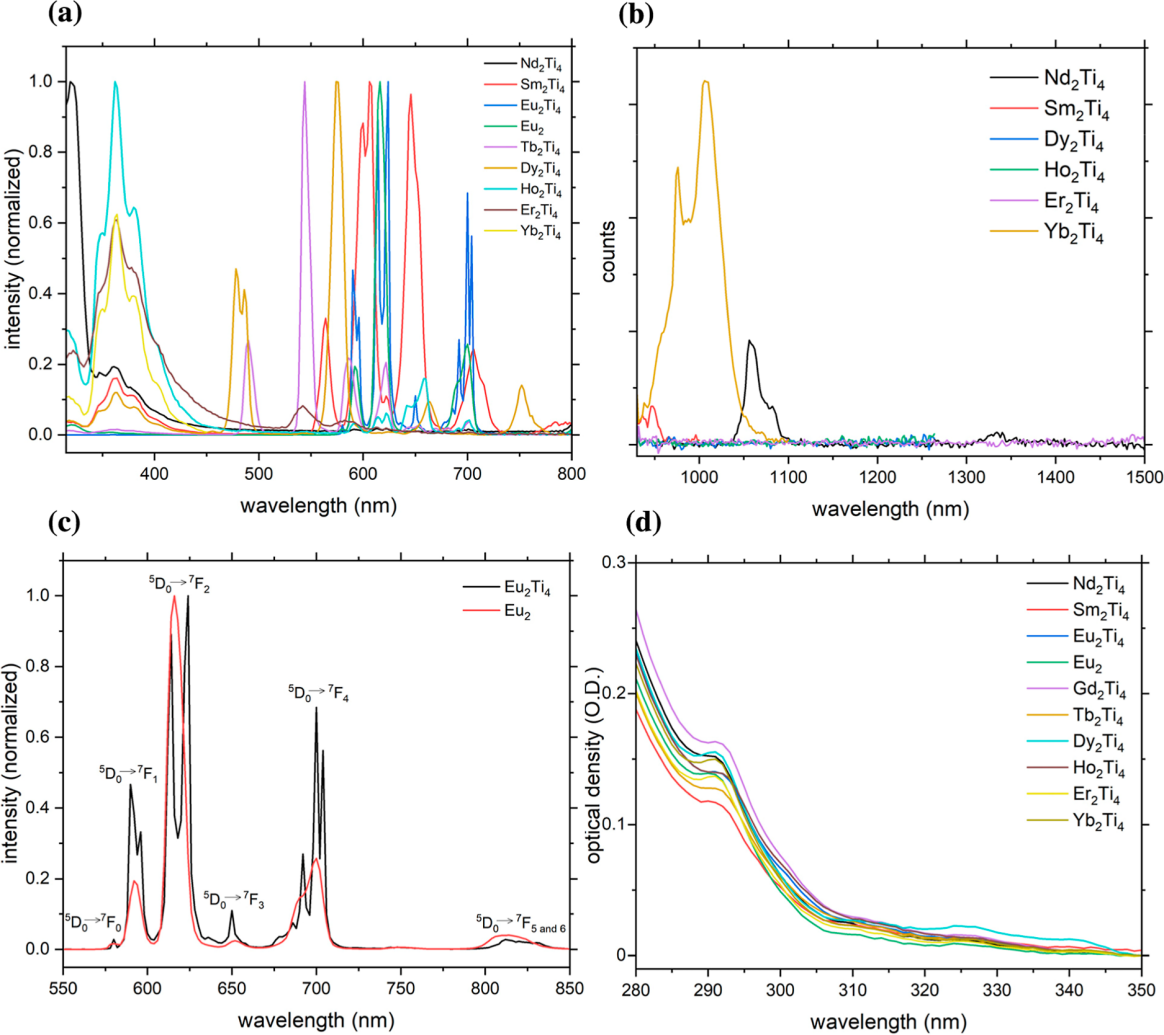
(a) The normalized visible-emission spectra of all compounds in solution (0.1 mg/mL, DCM) showing the respective characteristic lanthanide emission and residual ligand emission between 330 and 450 nm for Ln = Nd, Sm, Tb, Dy, Ho, Er, and Yb. (b) NIR-emission spectra for the compounds containing NIR-emitting lanthanides (**Nd**_**2**_ **Ti**_**4**_, **Sm**_**2**_ **Ti**_**4**_, **Dy**_**2**_ **Ti**_**4**_, **Ho**_**2**_ **Ti**_**4**_, **Er**_**2**_ **Ti**_**4**_, **Yb**_**2**_ **Ti**_**4**_). (c) Comparison of the normalized emission spectra of **Eu**_**2**_ and **Eu**_**2**_ **Ti**_**4**_, including the assignment of the observed electronic transitions. (d) Absorption spectra of the novel compounds in solution (0.05 mg/mL, DCM).

For most of the compounds studied the intramolecular energy transfer between the ligand and the lanthanide does not seem to go to completion and residual ligand fluorescence of varying intensity is observed in the emission spectra of the **Ln**_**2**_ **Ti**_**4**_ cages (Ln = Nd, Sm, Tb, Dy, Ho, Er, Yb, Figures 3a and S9). For Ln = Eu the lanthanide emission is of much higher intensity relative to the residual ligand fluorescence which is why the latter cannot be seen in the normalized emission spectra in Figure 3a. The comparison of the room-temperature emission spectra of **Eu**_**2**_ and **Eu**_**2**_ **Ti**_**4**_ reveals some distinct differences when moving from the 8-coordinate dodecahedral geometry in **Eu**_**2**_ **Ti**_**4**_ to the 9-coordinate tricapped trigonal prismatic geometry in **Eu**_**2.**_ Both the ^5^D_0_ → ^7^F_1_ and ^5^D_0_ → ^7^F_2_ bands are split into two transitions in **Eu**_**2**_ **Ti**_**4**_ and the respective ^5^D_0_ → ^7^F_4_ band shows an even more complex substructure compared to **Eu**_**2**_ due to the ligand field-induced Stark splitting of the ^7^F_4_ state (Figure 3c). This indicates a higher symmetry of the coordination environment of Eu^3+^ in **Eu**_**2**_ **Ti**_**4**_ compared to that in **Eu**_**2**_.^17^ The asymmetry ratio *R*, which can be derived from the ratio of the integrated intensities of the ^5^D_0_ → ^7^F_2_ and the ^5^D_0_ → ^7^F_1_ (magnetic dipole) transitions, lies at around 2.8 for **Eu**_**2**_ **Ti**_**4**_ and **Eu**_**2**_ as their Eu^3+^ coordination geometries are far off from being centrosymmetric. The presence of only one ^5^D_0_ → ^7^F_0_ transition suggests that the two Eu^3+^ centers in each compound are identically coordinated, which lies in agreement with the crystallographic data.^18^ The generally higher peak resolution in the emission spectrum of **Eu**_**2**_ **Ti**_**4**_ compared to that of **Eu**_**2**_ could further indicate a reduction of thermal vibrations within the structure. This assumption is supported by comparing the **Eu**_**2**_ spectrum to those of structurally related Eu_2_(phen)_2_(carboxylate)_6_ compounds, which confirms the presence of broad peaks at room temperature and a much better resolution of the peak splitting at 77 K, as a result of the reduction in quenching.^19^

### Investigation of the Intramolecular Energy Transfer Process

Regarding the variation in residual ligand emission in the luminescence spectra, we proceeded to investigate the intramolecular energy transfer process in more detail. The literature suggests two main sensitization mechanisms, one involving an intermediate ligand triplet state (Figure 1).^9^ In order to determine the sensitization pathway, we first attempted to measure the transient absorption (TA) spectra of a few selected compounds (**Eu**_**2**_ **Ti**_**4**_, **Gd**_**2**_ **Ti**_**4**_, and **Yb**_**2**_ **Ti**_**4**_) as well as that of free phenanthroline (Figure S12). This would allow the determination of the sensitization efficiency η_sens_ as^8^

2 $$\eta_{sens}=\phi^{SET}+\phi^{ISC}\phi^{TET}=\frac{k_{SET}}{\sum k_{singlet}}+\frac{k_{ISC}}{\sum k_{singlet}}\frac{k_{TET}}{\sum k_{triplet}}$$

Where ϕ represents a quantum yield and *k* represents a rate. The lifetime quenching of either the excited singlet or triplet state may also be used to calculate the transfer efficiency of that state if an appropriate reference compound is used (i.e., one in which other rate constants are not significantly perturbed) using the following equation^6b^

3 $$\eta_{sens}=1-\frac{\tau_{da}}{\tau_{d}}$$

Where τ_da_ is the lifetime of the donor–acceptor compound (the **Ln**_**2**_ **Ti**_**4**_ compounds) and τ_d_ is that of the donor only (**Gd**_**2**_ **Ti**_**4**_, where no ligand-to-Ln^3+^ energy transfer takes place).

We first attempted to measure the respective lifetimes (τ_da_ and τ_d_) via TA spectroscopy. However, for all compounds, including the free phenanthroline ligand, only one weak, short-lived signal was seen in the detectable spectral range (400–800 nm). This appears at around 550 nm upon pumping at 320 nm with no sign of a delayed onset, which suggests a singlet state rather than a triplet (which would appear with a slight delay due to the required change in spin during ISC). A more in-depth interpretation of the TA spectra was complicated by system limitations such as the inability to pump or probe as deep into the UV as required as well as insufficient sample concentration and aggregation (Figure S13). Therefore, the exact sensitization pathway or efficiencies could not be determined using this technique.

An alternative approach to estimating η_sens_ is to compare the normalized intensities of the lanthanide-centered emissions (Figure 4). This approximation takes both the intrinsic PLQY Φ_intrinsic_ and the amount of ligand-centered absorption into account, but relies on the careful calibration of the detectors used in the measurements. When comparing the relative efficiencies shown in Figure 4 it should be noted that for all lanthanides (apart from Eu) Φ_intrinsic_ was determined based on radiative lifetimes obtained from the literature, which only provides a rough estimate (Table 1). Despite this, the sensitization process appears to be significantly more efficient for Ln = Eu (**Eu**_**2**_ **Ti**_**4**_ and **Eu**_**2**_) than for the remaining lanthanides. The observed difference between the two Eu-containing compounds could result from the aforementioned change in the coordination geometry of the Eu^3+^ centers (dodecahedral for **Eu**_**2**_ **Ti**_**4**_ and tricapped trigonal prismatic for **Eu**_**2**_). A lower symmetry around the Eu^3+^ would make the 4f–f transition less symmetry forbidden which would increase their oscillator strength and therefore the molar extinction coefficient ε. This would increase the efficiency *E* of Förster resonance energy transfer (FRET) according to

4 $$E=\frac{1}{1+{\left(\frac{r}{R_{0}}\right)}^{6}}\left[R_{0}^{6}\propto J\propto \varepsilon_{A}\right]$$

**Figure 4:**
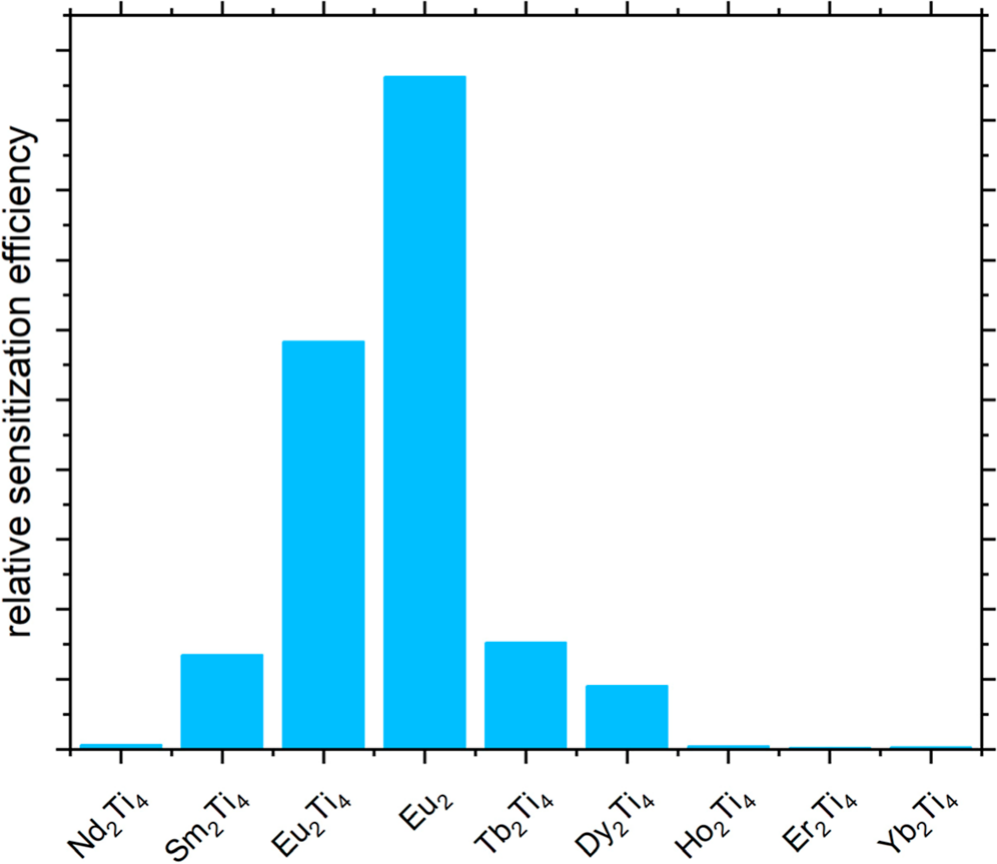
The relative sensitization efficiencies for **Ln**_**2**_ **Ti**_**4**_ and **Eu**_**2**_ which were determined from the lanthanide-centered emission intensities, normalized by Φ_intrinsic_ and the absorption intensity (300 nm excitation with 13 nm bandgap) as η_sens_(rel) = *I*_em_/(*I*_abs_ * Φ_intrinsic_).

**Table 1:** Emission Lifetimes and Estimated Intrinsic PLQYs of the Novel Compounds^e^

compound	transition	τ_exp_ [μs]^a^	τ_rad_ [ms]^b^	Φ_intrinsic_ [%]^c^	*k*_nr_ [ms^–1^]^d^
Nd_2_Ti_4_	^4^F_3/2_ → ^4^I_11/2_	3.5	0.42	8.3	
Sm_2_Ti_4_	^4^G_5/2_ → ^6^H_7/2_	20.8	6.26	3.3	
Eu_2_Ti_4_	^5^D_0_ → ^7^F_2_	2626	3.94	67	0.13
Eu_2_	^5^D_0_ → ^7^F_2_	1530	2.23	68	0.21
Tb_2_Ti_4_	^5^D_4_ → ^7^F_5_	36.6	9.00	4.1	
Dy_2_Ti_4_	^4^F_9/2_ → ^6^H_13/2_	6.3	1.85	3.4	
Ho_2_Ti_4_	^5^F_5_ → ^5^I_8_	2.4	0.80	3.0	
Er_2_Ti_4_	^4^I_13/2_ → ^4^I_15/2_	4.5	0.70	6.4	
Yb_2_Ti_4_	^2^F_5/2_ → ^2^F_7/2_	16.7	1.30	12.8	

a For biexponential fits the average lifetime was calculated as $\tau =\frac{\alpha_{1}\tau_{1}^{2}+\alpha_{2}\tau_{2}^{2}}{\alpha_{1}\tau_{1}+\alpha_{2}\tau_{2}}$ .

b Obtained from $\frac{1}{\tau_{rad}}=A_{MD,0}{\cdot n}^{3}\left(\frac{I_{tot}}{I_{MD}}\right)$ for Ln = Eu, with *n* = 1.42 (DCM). For all other cases the values are estimated from the literature.^21^

c $\Phi_{intrinsic}=\frac{\tau_{\exp}}{\tau_{rad}}$ .

d $k_{nr}=\frac{1-\Phi_{intrinsic}}{\tau_{\exp}}$ (estimation).

e The measurements were carried out in solution (DCM). Details on the exponential fitting are given in the Supporting Information (Figure S11, Tables S6 and S7).

Where *R*_0_ is the Förster distance of the donor–acceptor pair, *J* is the overlap integral of the donor emission with the acceptor absorption and ε_A_ is the molar extinction coefficient of the acceptor.^6b^

For Ln = Sm, Tb and Dy, η_sens_ still indicates some degree of efficient energy transfer, whereas the NIR-emitters **Nd**_**2**_ **Ti**_**4**_, **Ho**_**2**_ **Ti**_**4**_, **Er**_**2**_ **Ti**_**4**_, and **Yb**_**2**_ **Ti**_**4**_ are only poorly sensitized. This decrease could result from a combination of energy-level mismatch and energy back-transfer onto the phenanthroline ligand.

An attempt to further estimate the contribution of the ligand singlet and triplet states to the energy transfer efficiency was made by quantifying the residual phenanthroline fluorescence. The resulting normalized emission intensities are shown and discussed in Figure S10. However, the results were inconclusive.

In view of the electronic structures of the Ln^3+^ ions, some comments on the mechanism of the intramolecular energy transfer process can be made (Figure 5). For the lanthanides (Ln = Sm, Eu, Ho, Er) in which the main emissive state (highlighted in blue) lies around 2000–3000 cm^–1^ below the T_1_ state of phenanthroline, we can assume participation of the triplet state in the energy transfer process. For Ln = Nd this might also be the case, as the ^2^P_J_ states lie close enough to the T_1_ state to accept energy and then pass it down to the emissive ^4^F_3_ state via IC. However, for all of these lanthanides an additional contribution of the excited singlet state of phenanthroline (S_1_) to their sensitization is also likely. For Ln = Tb and Dy the emissive ^5^D_4_ and ^4^F_9/2_ states, respectively, appear to lie slightly above the phenanthroline triplet state, which suggests mainly sensitization from the excited singlet state. The actual energy of these states is likely to deviate from the theoretical prediction shown in Figure 5, however, and a contribution of the T_1_ state cannot be excluded. The only excited state in Yb^3+^ (^2^F_5/2_) lies too far below the ligand singlet or triplet states to allow efficient sensitization, explaining the low emission intensity observed for **Yb**_**2**_ **Ti**_**4**_.

**Figure 5:**
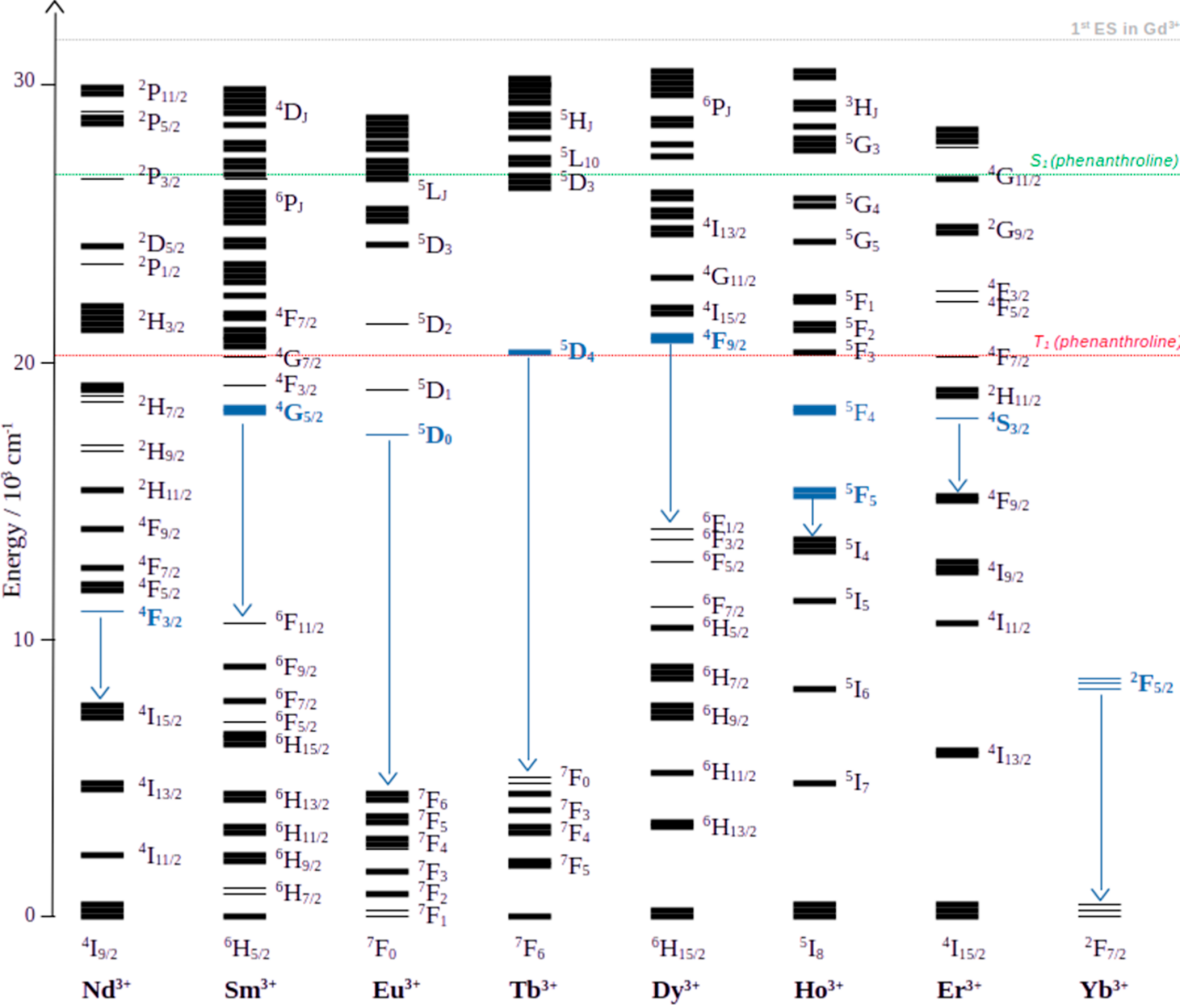
A simplified overview of the electronic structure of lanthanide­(III) ions. The main emissive states and the distance to the nearest accepting state are highlighted in blue. The energy of the S_1_ and T_1_ states in phenanthroline were determined from the room-temperature emission spectrum of **Gd**_**2**_ **Ti**_**4**_ (Figure S8). Adapted from ref 20. Copyright 2009 American Chemical Society.

The second part of the phen → Ln^3+^ energy transfer process to be analyzed is the emission of the lanthanide ion upon sensitization. The radiative lifetimes τ_exp_ of all **Ln**_**2**_ **Ti**_**4**_-type compounds were measured and Φ_intrinsic_ was estimated based on the results (Table 1, Figure S11). Since τ_exp_ only depends on the electronic structure and the coordination environment of the lanthanide ion, as well as external factors such as the refractive index of the sample, no conclusions on the sensitization process or overall brightness of the compounds can be drawn from these measurements.

In most cases (Ln = Nd, Sm, Dy, Ho, Er, Yb) the low lifetimes result from a small energy gap between the lowest emitting level of the excited state and the highest level of the ground state (Figure 5), which increases *k*_nr_, the rate of nonradiative decay processes (IC or vibrational quenching, Figure 1). The relation between τ and the two rate constants *k*_r_ (radiative processes) and *k*_nr_ (nonradiative processes) is given below^6b^

5 $$\tau =\frac{1}{k_{r}+\Sigma k_{nr}}$$

With an increasing energy separation of the two levels (Eu, Tb) τ_exp_ increases. For Eu^3+^ this effect is most pronounced, which leads to an increase in τ_exp_ by 2 orders of magnitude. It should be noted that a similar result would be expected for Tb^3+^ as well, given the large gap between the ^5^D_4_ and ^7^F_0_ states. In some cases (Nd, Sm, Tb, Ho) the radiative decay was fitted biexponentially, which could result from cross-relaxation between the two lanthanide centers in the molecule or potentially trace amounts of the respective **LnTi**_**6**_ compounds.

### Quantifying the Impact of the Metal-Oxo Core

In order to quantify the impact of the rigid titanium-oxo core on both the sensitization efficiency and the lifetime of the lanthanide center, we measured the emission properties of **Eu**_**2**_, as this compound includes the same Ln-phen unit as the **Ln**_**2**_ **Ti**_**4**_ cages but lacks the metal-oxo framework (Figure 2). A thorough comparison of the respective lifetimes (Figure 6, Table S7) shows a significant increase of τ_exp_ from 1.5 ms (**Eu**_**2**_) to 2.6 ms (**Eu**_**2**_ **Ti**_**4**_) upon coordination of the Eu^3+^ to the Ti_4_O_6_-core, which can be attributed to the reduction of nonradiative decay processes (*k*_nr_, Table 1) resulting from the coordination. It should be noted that the different natural lifetimes τ_rad_ for **Eu**_**2**_ **Ti**_**4**_ and **Eu**_**2**_ could also contribute to some extend to this increase.

**Figure 6:**
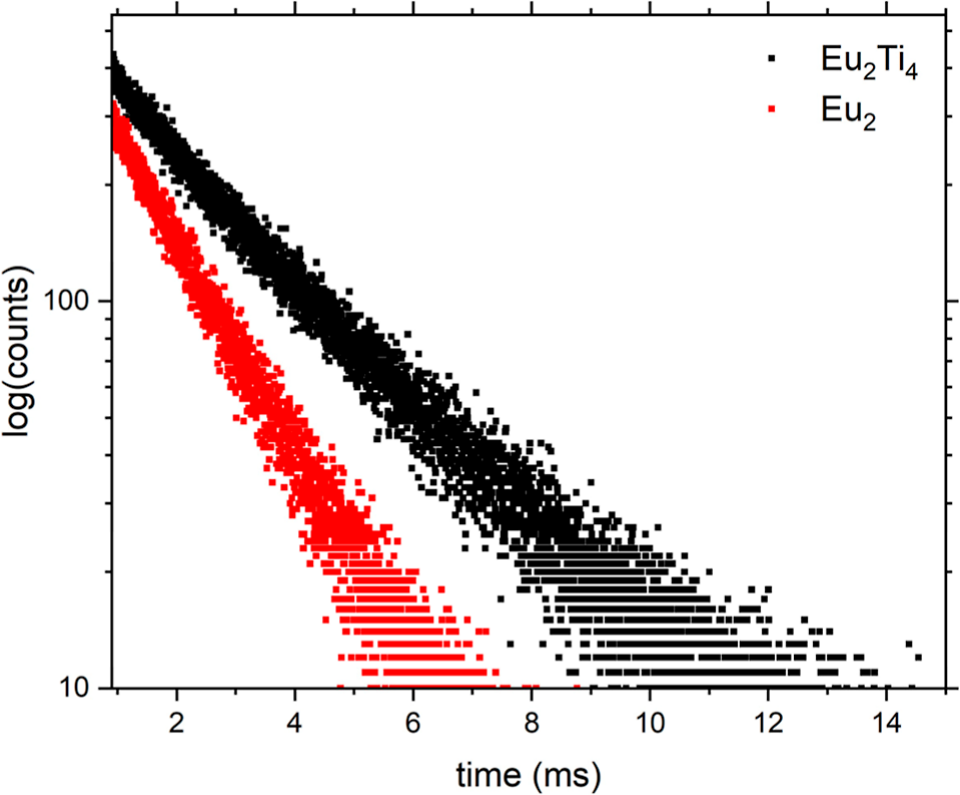
Radiative decay curves of **Eu**_**2**_ **Ti**_**4**_ and **Eu**_**2**_ showing the increased lifetime of the Eu^3+^ center in **Eu**_**2**_ **Ti**_**4**_ upon coordination to the metal-oxo core. Details of the exponential fits are given in Table S7.

These measurements provide strong evidence for the positive impact of the metal-oxo core on the emission properties of a sensitized lanthanide ion. Compared to the literature, the lifetime of Eu^3+^ in **Eu**_**2**_ is still considerably longer than that of mononuclear Eu-phen compounds such as [EuCl_2_Phen_2_(H_2_O)_2_]­Cl­(H_2_O) (0.27 ms), since the first coordination sphere of the lanthanide in the latter compounds is more likely to contain highly oscillating O–H quenchers.^22^

## Conclusion

In this work the synthesis and photophysical properties of a series of novel **Ln**_**2**_ **Ti**_**4**_ cages were investigated. Both stoichiometry and water content were found to have an impact on the size of the titanium-oxo core of the product. Compared to the dinuclear **Eu**_**2**_ cage, the rigid bonding of the Ti_4_O_6_-core to the Eu^3+^ ion in **Eu**_**2**_ **Ti**_**4**_ increases the lifetime of the excited ion significantly by around 1 ms. A rough estimate of the relative sensitization efficiencies was obtained by comparing the normalized relative emission intensities of the compounds, which proved Eu^3+^ to be the most efficient acceptor for the phenanthroline antenna ligand. The analysis of the nonemissive **Gd**_**2**_ **Ti**_**4**_-compound showed room-temperature phosphorescence which indicated the position of the ligand triplet state in the **Ln**_**2**_ **Ti**_**4**_ compounds.^20^

In conclusion, this work presents an in-depth analysis of the intramolecular energy transfer processes of lanthanide-containing POTs involving an antenna ligand. Fundamental photophysical measurements of the **Ln**_**2**_ **Ti**_**4**_ cages containing a large range of Ln^3+^ ions provide crucial information regarding the future design of sensitized Ln-POTs. Perhaps most importantly, our design strategy of incorporating Ln^3+^ ions into a rigid polyoxotitanate core, rather than using more traditional coordination compounds, has been validated, resulting in a significant increase in the lifetime of the Eu^3+^ excited state compared to a benchmark coordination compound.

## Supplementary Material



## Notes

IR spectra, crystallographic data (sc- and pXRD), photophysical measurements (excitation spectra, lifetime fitting, TA spectra) and DOSY data for **Eu**_**2**_ **Ti**_**4**_ (PDF)

## References

[1] b Bünzli, J.-C. G. Lanthanides and Actinides: Synthesis, Reactivity, Properties and Applications; Wold Scientific, 2022; pp 633–685.

[2] Jia J.-H., Li Q.-W., Chen Y.-C., Liu J.-L., Tong M.-L. (2019). Luminescent
single-molecule magnets based on lanthanides: Design strategies, recent
advances and magneto-luminescent studies. Coord.
Chem. Rev..

[3] He P., Wang H. H., Liu S. G., Hu W., Shi J. X., Wang G., Gong M. L. (2009). An efficient europium­(III)
organic complex as red phosphor applied in LED. J. Electrochem. Soc..

[4] Wang T., Wang S., Liu Z., He Z., Yu P., Zhao M., Zhang H., Lu L., Wang Z., Wang Z., Zhang W., Fan Y., Sun C., Zhao D., Liu W., Bünzli J.-C. G., Zhang F. (2021). A hybrid erbium­(III)-bacteriochlorin near-infrared
probe for multiplexed biomedical imaging. Nat.
Mater..

[5] Zhu Z., Guo M., Li X.-L., Tang J. (2019). Molecular magnetism of lanthanide:
Advances and perspectives. Coord. Chem. Rev..

[6] Weissman S. I. (1942). Intramolecular energy transfer The
fluorescence of
complexes of europium. Chem. Phys..

[7] Müller R., Okokhere-Edeghoghon B., Janowicz N. J., Bond A. D., Kociok-Kohn G., Cox L. M. R., Garzon D., Waine T. W., Truckell I. G., Gage E., Thompson A. J., Busko D., Howard I. A., Saavedra M. S., Richards B. S., Breiner B., Cameron P., Wright D. S. (2024). Transparent, sprayable plastic films for luminescent
down-shifted-assisted plant growth. Adv. Mater.
Technol..

[8] Latva M., Takalo H., Mukkala V. M., Matachescu C., Rodriguez-Ubis J.-C., Kankare J. (1997). Correlation between the lowest triplet
state energy level of the ligand and lanthanide­(III) luminescence
quantum yield. J. Lumin..

[9] Kleinerman M. (1969). Energy migration
in lanthanide chelates. J. Chem. Phys..

[10] Amoroso A. J., Pope S. J. A. (2015). Using lanthanide ions in molecular bioimaging. Chem. Soc. Rev..

[11] Shu X.-P., Luo W., Wang H.-Y., Fu M.-Y., Zhu Q.-Y., Dai J. (2020). Eu-phen bonded
titanium oxo-clusters, precursors for a facile preparation of high
luminescent materials and films. Inorg. Chem..

[12] Sheldrick G. M. (2015). Crystal
structure solution with ShelXT. Acta Crystallogr.,
Sect. A: Found. Crystallogr..

[13] Sheldrick G. M. (2015). Crystal
structure refinement with SHELXL. Acta Crystallogr.,
Sect. C: Struct. Chem..

[14] Liu W.-D., Li G.-J., Xu H., Deng Y.-K., Du M.-H., Long L.-S., Zheng L.-S., Kong X.-J. (2023). Circularly polarized luminescence and performance modulation
of chiral europium-titanium (Eu_2_Ti_4_)-oxo clusters. Chem. Commun..

[15] Lu W.-M., Shao Z.-P., Hu J.-B., Luo X.-Y., Dong N., Gu J.-M. (1996). Synthesis, Spectra
and Crystal Structure of *Bis*(1,10-phenanthroline)­di­(μ-α-methacrylato)
Lanthanide­(III) Dimers. J. Coord. Chem..

[16] Lu W., Luo X., Wu B., Mao J., Jiang X. (1999). A dihomonuclear complex:
di-μ-methacrylato-*O:O*′-bis­[(1,10-phenanthroline-*N*,*N*′)­bis­(methacrylato-*O*,*O*′)­ytterbium­(III)]. Acta Crystallogr., Sect. C: Cryst. Struct. Commun..

[17] Serna-Gallén P., Beltrán-Mir H., Cordoncillo E. (2023). Practical
guidance for easily interpreting
the emission and physicochemical parameters of Eu^3+^ in
solid-state hosts. Ceram. Int..

[18] Tanner P. A. (2013). Some misconceptions
concerning the electronic spectra of tri-positive europium and cerium. Chem. Soc. Rev..

[19] Barja B., Aramendia P., Baggio R., Garland M. T., Peña O., Perec M. (2003). Europium­(III) and terbium­(III) *trans*-2-butenoates:
syntheses, crystal structures, and properties. Inorg. Chim. Acta.

[20] Moore E. G., Samuel A. P. S., Raymond K. N. (2009). From Antenna
to Assay: Lessons Learned
in Lanthanide Luminescence. Acc. Chem. Res..

[21] Carnall, W. T. Handbook of the Physics and Chemistry of Rare Earths; North Holland Publ. Co: Amsterdam, 1979, Vol. 3, pp 172–208, Chapter 24.

[22] Puntus L. N., Lyssenko K. A., Antipin M. Yu., Bünzli J.-C. G. (2008). Role
of inner- and outer-sphere bonding in the sensitization of Eu^III^-luminescence deciphered by combined analysis of experimental
electron density distribution function and photophysical data. Inorg. Chem..

