# Supplementary material for: Understanding the Photophysical Properties of the Polyoxotitanates Ln2Ti4O6(phen)2(met)10
Source: Inorg Chem. 2025 Jul 31;64(32):16461–9. doi: 10.1021/acs.inorgchem.5c02216 (PMC12365869; doi:10.1021/acs.inorgchem.5c02216)
Supplement: Supplementary file 1 [file ic5c02216_si_001.pdf]

Supplementary Information for

Understanding the Photophysical Properties of the Polyoxotitanates $Ln_2Ti_4O_6(phen)_2(met)_{10}$

Rosa Müller,[a] Alasdair Tew,[b] Andrew D. Bond,[a] Akshay Rao,[b] Hugo Bronstein[a] and Dominic S. Wright[a,]*

[a] Yusuf Hamied Department of Chemistry, Cambridge University, Cambridge CB2 1EW; E-mail: dsw1000@cam.ac.uk
[b] The Cavendish Laboratory, Department of Physics, Cambridge University, Cambridge CB3 0HE.

1. IR data

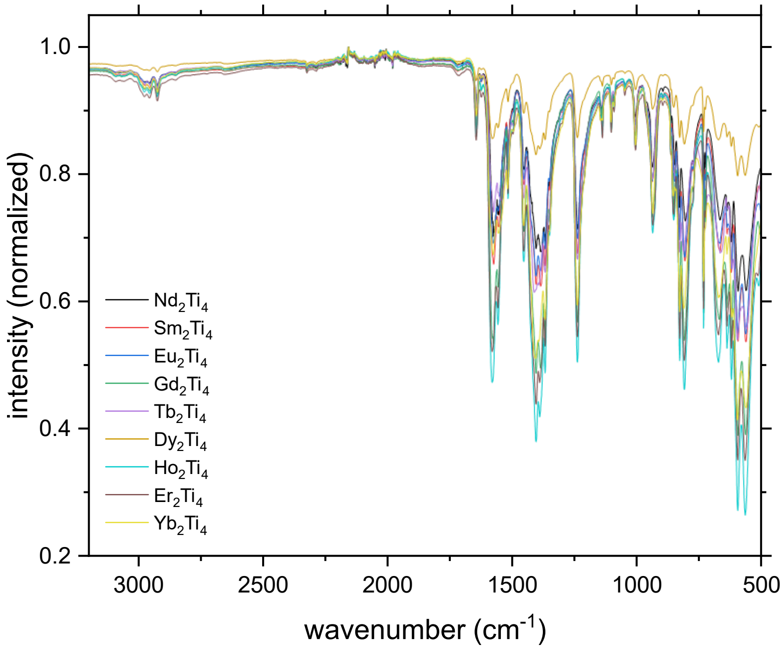

Figure S1. FT-IR spectra of the series of $\mathbf{Ln_2Ti_4}$ compounds.

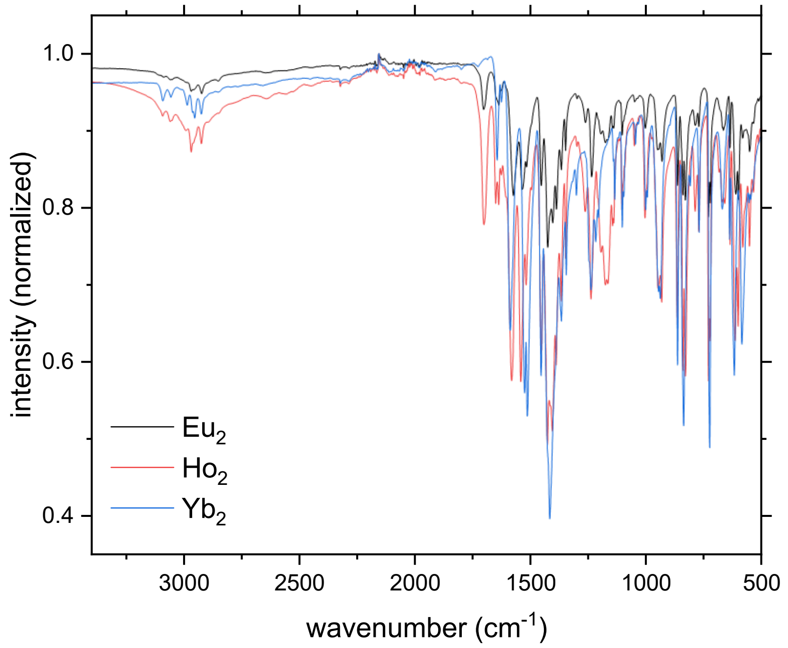

Figure S2. FT-IR spectra of $[Ln_2(phen)_2(met)_6]$ for Ln = Eu, Ho and Yb.

2. Crystallographic Data

2.1 Powder diffraction data

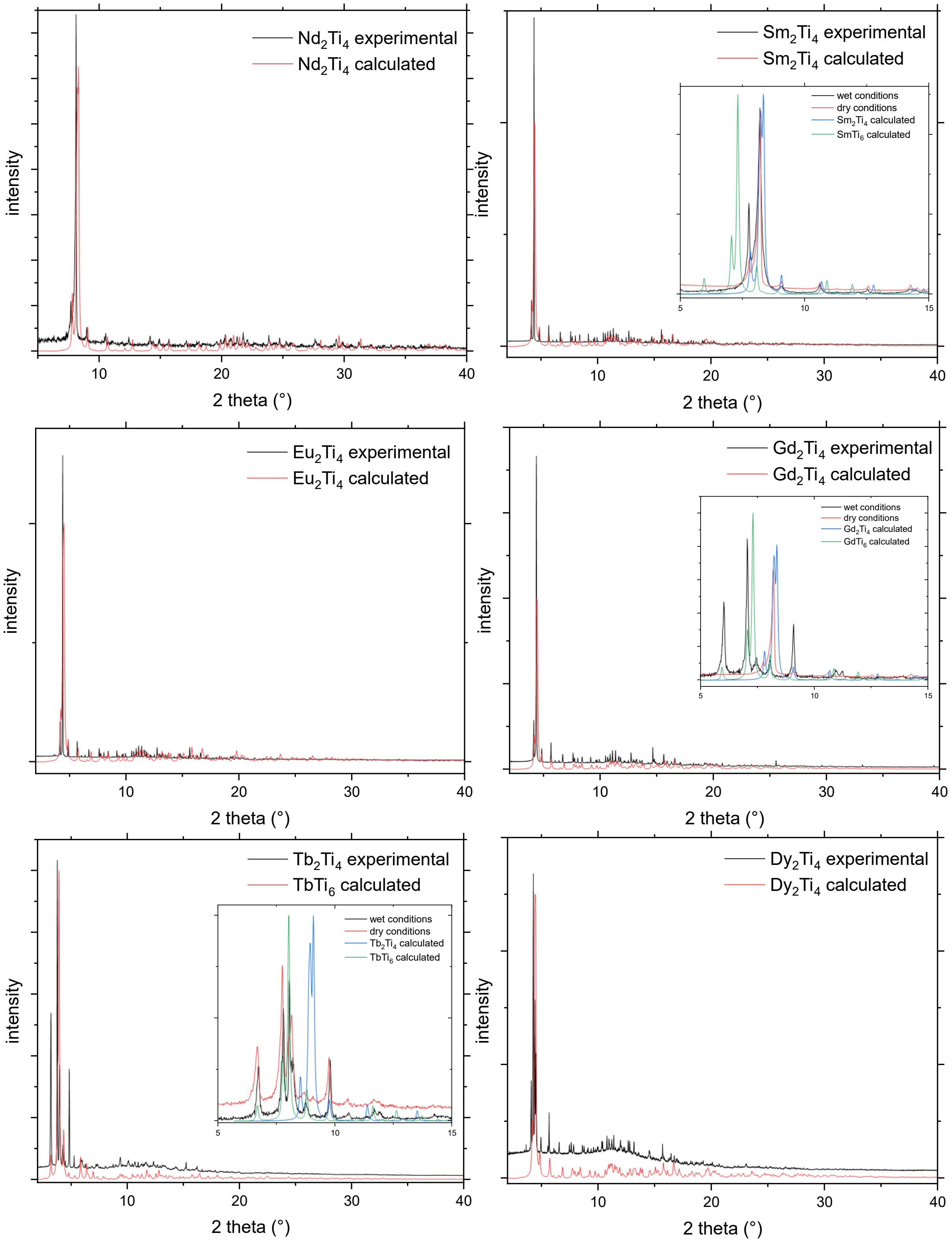

Figure S3. Comparison of the experimental pXRD patterns (room temperature) with those calculated from the crystal structures of **Nd_2Ti_4, Sm_2Ti_4, Eu_2Ti_4, Gd_2Ti_4, Tb_2Ti_4** and **Dy_2Ti_4** (determined at 180 K). In the cases where the **$LnTi_6$**-type compounds were detected as single-crystals (Ln = Sm, Gd, Tb,

determined at 220 K) the inserts show a comparison of the bulk reaction products from both wet and dry conditions with the respective calculated diffraction patterns. For Ln = Sm the **$SmTi_6$** compound could not be detected in the bulk product regardless of the reaction conditions, whereas for Ln = Gd the **$GdTi_6$** compound was the main product under wet reaction conditions only. For Ln = Tb the **$TbTi_6$** compound was the main component of the bulk product under both wet and dry conditions.

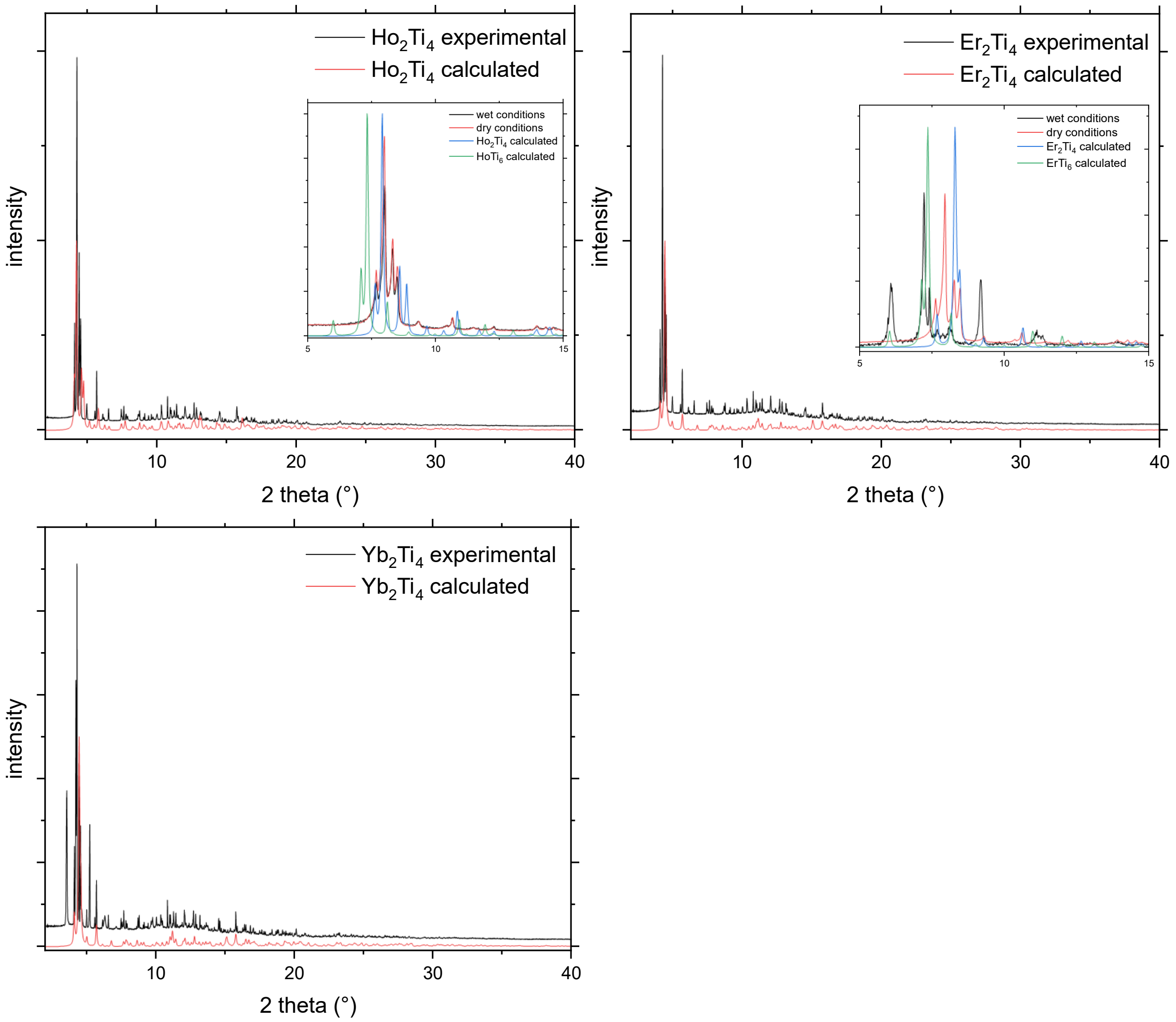

Figure S4. Comparison of the experimental pXRD patterns (room temperature) with those calculated from the crystal structures of **Ho_2Ti_4**, **Er_2Ti_4** and **Yb_2Ti_4** (determined at 180 K). In the cases where the **$LnTi_6$**-type compounds were detected as single-crystals (Ln = Ho, Er, determined at 220 K) the inserts show a comparison of the bulk reaction products from both wet and dry conditions with the respective calculated diffraction patterns. For Ln = Ho the **$HoTi_6$** compound could not be detected in the bulk product regardless of the reaction conditions, whereas for Ln = Er the **$ErTi_6$** compound was the main product under wet reaction conditions only.

*Comment on the potential contamination of **Ln_2Ti_4** with **$LnTi_6$***

ScXRD was used to identify and characterize the two components (**Ln_2Ti_4** with **$LnTi_6$**) present in the product mixture based on the different morphologies of the crystals (**Ln_2Ti_4**: clear blocks, **$LnTi_6$**: indistinct globules). In each case only trace amounts of the **$LnTi_6$** crystal type were observed (apart from Tb where the quantity is more variable), and their formation was found to be highly dependent on the amount of moisture in the reaction mixture. This does not affect the bulk analysis of the **Ln_2Ti_4** samples obtained under strictly dry conditions (elemental analysis and pXRD), apart from the case of

Tb (Figure S3, S4). The purity of the compounds is therefore not questionable. For **Tb_2Ti_4** the seemingly greater moisture content leads to variable amounts of **$TbTi_6$**.
The photophysical studies were done very carefully regarding the potential **$LnTi_6$** contamination and the phase purity of every **Ln_2Ti_4** sample used for optical analysis was confirmed using EA and pXRD. The only issue is that of **Tb_2Ti_4** in which variable amounts of the **$TbTi_6$** component are present. Since the main purpose of this study was to analyze the energy transfer between phenanthroline and the Ln^{3+} ion within a polyoxotitanate framework, and the same Ln-phen unit is present in both **Tb_2Ti_4** and **$TbTi_6$**, this is not considered to impact the statements made in this study (i.e., where we analyze the position of the energy states in phenanthroline relative to those in the Ln^{3+} ions). One indicator of the potential presence of both Tb species in the sample is the biexponential fit of the luminescence decay (Figure S11).

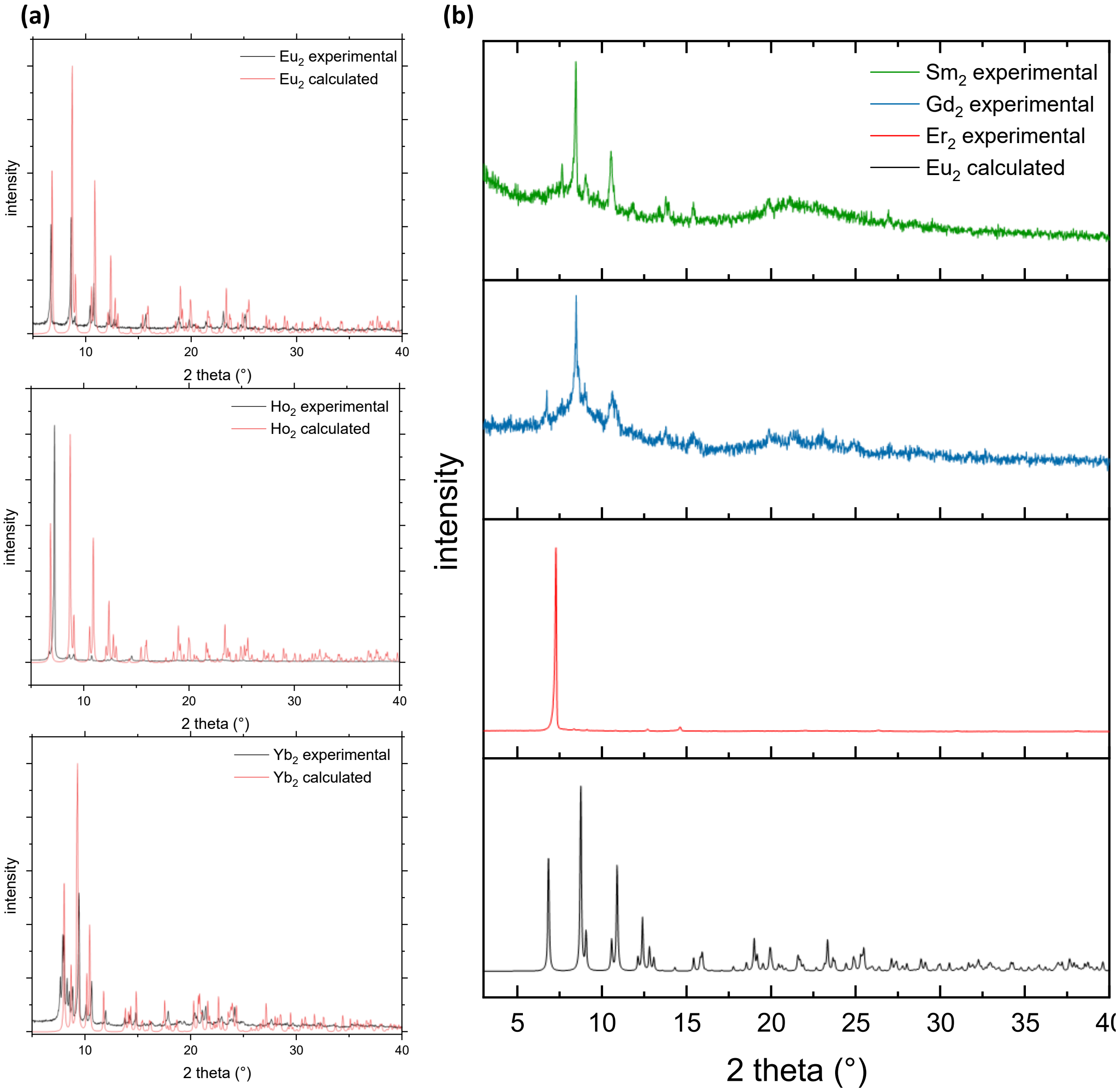

Figure S5. (a) Experimental vs calculated pXRD patterns for **Eu_2**, **Ho_2** and **Yb_2**. (b) The experimental pXRD patterns for **Sm_2**, **Gd_2** and **Er_2** are compared to the calculated pattern for **Eu_2**, since no single-crystal data was obtained for these compounds. No further analysis was carried out on these dimeric compounds, apart from for **Eu_2**.

2.2 Single-crystal diffraction data

Crystal structures of $LnTi_4$

The crystal structures of $\mathbf{Ln_2Ti_4}$ [Ln = Nd, Sm, Gd, Tb, Dy, Ho, Er, Yb] are isomorphous with the previously published $\mathbf{Eu_2Ti_4}$ compound (CSD: NUYFIZ).[1] Since the *a* and *c* lengths of the unit cell are similar, care was taken to ensure that the structures are reported in a consistent cell setting with comparable coordinate sets. As a consequence, some unit cells are not reduced ($a > c$).

Refinement was generally straightforward. Some attention was needed to distinguish between the $C{=}CH_2$ and $C{—}CH_3$ groups of the methacrylate ligands. In most cases, the C—C bond lengths were clearly different and the H atoms were placed in idealized positions on that basis. In some cases, the C—C bond lengths were similar, which possibly indicates disorder (i.e., the methacrylate ligand could be present in either orientation). Where there was doubt, the H atoms were placed by comparison to the other structures, so as to yield a fully consistent arrangement across the entire series.

Crystal structures of $LnTi_6$

Where suitable crystals of $\mathbf{LnTi_6}$ [Ln = Sm, Tb, Ho] were found in the same reaction mixtures as $\mathbf{Ln_2Ti_4}$, the two could generally be distinguished by sight: $\mathbf{Ln_2Ti_4}$ had a more clearly defined block-like appearance, while $\mathbf{LnTi_6}$ has a less well-defined shape. Plunging crystals of $\mathbf{LnTi_6}$ directly into the N_2 cryostream at 180 K consistently yielded sub-optimal diffraction patterns, while the patterns at 220 K looked very good. Hence, all $\mathbf{LnTi_6}$ structures were determined at 220 K.

This structure type was also observed for Er and Gd, but collected single-crystal X-ray data yielded unsatisfactory refinements. We did not pursue re-determination for these lanthanides since the structure type is sufficiently well established by the three structures that are presented.

In the structure, the complex is situated on a crystallographic mirror plane such that one methacrylate ligand is positioned with its CH_2 and CH_3 groups related by the mirror symmetry. The H atoms of these groups were included as 50% CH_2 and 50% CH_3. The CH_2 and CH_3 groups were distinguished as described for $\mathbf{Ln_2Ti_4}$. Some differences in the ligand orientation are seen for the three different structures.

The structure contains substantial void space (ca. 25% of the unit-cell volume) in continuous channels along the *a* axis. Some electron density is seen in the Fourier map indicative of solvent molecules (most likely MeCN). However, it was difficult to refine discrete molecules, so the *SQUEEZE*[2] algorithm has been applied. For the three structures, *SQUEEZE* corrects for 188–216 electrons in the unit cell, corresponding to approx. 8–10 MeCN molecules.

Crystal structures of Ln_2

The crystal structures obtained for $\mathbf{Eu_2}$ and $\mathbf{Ho_2}$ are isomorphous. Both contain an additional isolated methacrylate molecule, forming a hydrogen bond to the $\mathbf{Ln_2}$ complex. The $\mathbf{Yb_2}$ structure contains disordered MeCN molecules in channels along the *a* axis. Refinement of these structures was straightforward.

Table S1. Crystal and refinement data for **Ln_2Ti_4** (Ln = Nd, Sm, Gd)

	**Nd_2Ti_4**	**Sm_2Ti_4**	**Gd_2Ti_4**
CCDC number	2430477	2430476	2430485
Cambridge data number	DW_B1_0603	DW_B1_0643	DW_B1_0644
Chemical formula	$C_{64}H_{66}N_4Nd_2O_{26}Ti_4$	$C_{64}H_{66}N_4O_{26}Sm_2Ti_4$	$C_{64}H_{66}Gd_2N_4O_{26}Ti_4$
Formula weight	1787.28	1799.50	1813.30
Temperature / K	180(2)	180(2)	180(2)
Crystal system	monoclinic	monoclinic	monoclinic
Space group	$P\ 2_1/n$	$P\ 2_1/n$	$P\ 2_1/n$
a / Å	12.9477(4)	12.9193(4)	12.8857(5)
b / Å	22.5969(7)	22.5732(6)	22.5773(8)
c / Å	13.1081(4)	13.1261(4)	13.1134(5)
alpha / °	90	90	90
beta / °	111.856(2)	112.0225(13)	112.042(2)
gamma / °	90	90	90
Unit-cell volume / $Å^3$	3559.5(2)	3548.67(18)	3536.2(2)
Z	2	2	2
Calc. density / g cm^{-3}	1.668	1.684	1.703
F(000)	1788	1796	1804
Radiation type	Cu Kα	Cu Kα	Cu Kα
Absorption coefficient / mm^{-1}	15.260	16.569	16.288
Crystal size / mm^3	0.12 x 0.08 x 0.04	0.16 x 0.14 x 0.06	0.12 x 0.08 x 0.03
2-Theta range / °	9.08-133.24	9.08-136.60	7.83-136.84
Completeness to max 2-theta	0.998	0.996	0.997
No. of reflections measured	62686	65233	57644
No. of independent reflections	6275	6486	6484
R(int)	0.0720	0.0597	0.1017
No. parameters / restraints	456 / 0	456 / 0	456 / 0
Final R1 values (I > 2[illegible](I))	0.0259	0.0271	0.0399
Final wR(F^2) values (all data)	0.0651	0.0700	0.1045
Goodness-of-fit on F^2	1.043	1.051	1.037
Largest difference peak & hole / e $Å^{-3}$	0.558, -0.500	0.962, -0.810	0.686, -1.411

Table S2. Crystal and refinement data for **Ln_2Ti_4** (Ln = Tb, Dy, Ho)

	**Tb_2Ti_4**	**Dy_2Ti_4**	**Ho_2Ti_4**
CCDC number	2430479	2430473	2430480
Cambridge data number	DW_B1_0601	DW_B1_0556	DW_B1_0557
Chemical formula	$C_{64}H_{66}N_4O_{26}Tb_2Ti_4$	$C_{64}H_{66}Dy_2N_4O_{26}Ti_4$	$C_{64}H_{66}Ho_2N_4O_{26}Ti_4$
Formula weight	1816.64	1823.80	1828.66
Temperature / K	180(2)	180(2)	220(2)
Crystal system	monoclinic	monoclinic	monoclinic
Space group	P 2_1/n	P 2_1/n	P 2_1/n
a / Å	12.8699(4)	12.8702(6)	13.2287(5)
b / Å	22.5669(7)	22.5972(8)	23.1404(9)
c / Å	13.1291(4)	13.1075(5)	11.9098(5)
alpha / °	90	90	90
beta / °	112.232(2)	112.177(2)	105.594(2)
gamma / °	90	90	90
Unit-cell volume / $Å^3$	3529.66(19)	3530.1(3)	3511.6(2)
Z	2	2	2
Calc. density / g cm^{-3}	1.709	1.716	1.729
F(000)	1808	1812	1816
Radiation type	Cu Kα	Cu Kα	Cu Kα
Absorption coefficient / mm^{-1}	14.018	15.489	8.395
Crystal size / mm^3	0.10 x 0.10 x 0.06	0.20 x 0.14 x 0.08	0.30 x 0.10 x 0.10
2-Theta range / °	9.08-136.60	9.09-136.70	10.33-138.18
Completeness to max 2-theta	0.998	0.998	0.987
No. of reflections measured	47494	58039	59641
No. of independent reflections	6465	6467	6449
R(int)	0.0690	0.1234	0.0521
No. parameters / restraints	456 / 0	456 / 0	456 / 18
Final R1 values (I > 2[illegible](I))	0.0322	0.0510	0.0304
Final wR(F^2) values (all data)	0.0855	0.1353	0.0792
Goodness-of-fit on F^2	1.022	1.046	1.071
Largest difference peak & hole / e $Å^{-3}$	0.822, -0.492	0.862, -1.455	0.527, -0.575

Table S3. Crystal and refinement data for **Ln_2Ti_4** (Ln = Er, Yb)

	**Er_2Ti_4**	**Yb_2Ti_4**
CCDC number	2430483	2430484
Cambridge data number	DW_B1_0589	DW_B1_0538
Chemical formula	$C_{64}H_{66}Er_2N_4O_{26}Ti_4$	$C_{64}H_{66}N_4O_{26}Ti_4Yb_2$
Formula weight	1833.32	1844.88
Temperature / K	180(2)	180(2)
Crystal system	monoclinic	monoclinic
Space group	P 2_1/n	P 2_1/n
a / Å	12.7827(6)	12.7365(5)
b / Å	23.0049(9)	23.0905(8)
c / Å	12.7369(5)	12.6638(4)
alpha / °	90	90
beta / °	109.848(2)	109.493(2)
gamma / °	90	90
Unit-cell volume / $Å^3$	3523.0(3)	3510.9(2)
Z	2	2
Calc. density / g cm^{-3}	1.728	1.745
F(000)	1820	1828
Radiation type	Cu Kα	Cu Kα
Absorption coefficient / mm^{-1}	8.595	9.114
Crystal size / mm^3	0.20 x 0.18 x 0.04	0.10 x 0.10 x 0.06
2-Theta range / °	7.69-136.98	9.35-136.96
Completeness to max 2-theta	0.997	0.996
No. of reflections measured	68216	62012
No. of independent reflections	6475	6438
R(int)	0.0758	0.0520
No. parameters / restraints	456 / 0	456 / 192
Final R1 values (I > 2[illegible](I))	0.0368	0.0276
Final wR(F^2) values (all data)	0.1026	0.0712
Goodness-of-fit on F^2	1.062	1.067
Largest difference peak & hole / e $Å^{-3}$	1.105, -0.632	0.932, -0.442

Table S4. Crystal and refinement data for **LnTi$_6$** (Ln = Sm, Tb, Ho)

	**SmTi$_6$***	**TbTi$_6$***	**HoTi$_6$***
CCDC number	2430474	2430482	2430481
Cambridge data number	DW_B1_0604	DW_B1_0605	DW_B1_0555
Chemical formula	$C_{56}H_{63}N_2O_{30}SmTi_6$	$C_{56}H_{63}N_2O_{30}TbTi_6$	$C_{56}H_{63}HoN_2O_{30}Ti_6$
Formula weight	1681.83	1690.40	1696.41
Temperature / K	220(2)	220(2)	220(2)
Crystal system	monoclinic	monoclinic	monoclinic
Space group	$P\ 2_1/m$	$P\ 2_1/m$	$P\ 2_1/m$
a / Å	15.0058(6)	14.9498(7)	14.9256(16)
b / Å	16.2304(7)	16.1805(8)	16.1810(18)
c / Å	18.3007(7)	18.3034(8)	18.315(2)
alpha / °	90	90	90
beta / °	99.544(2)	99.486(3)	99.501(5)
gamma / °	90	90	90
Unit-cell volume / Å^3	4395.4(3)	4367.0(4)	4362.6(8)
Z	2	2	2
Calc. density / g cm^{-3}	1.271	1.286	1.291
F(000)	1694	1700	1704
Radiation type	Cu Kα	Cu Kα	Cu Kα
Absorption coefficient / mm^{-1}	9.917	8.914	6.631
Crystal size / mm^3	0.14 x 0.12 x 0.08	0.14 x 0.12 x 0.08	0.30 x 0.10 x 0.10
2-Theta range / °	4.90-133.77	4.89-133.41	9.98-136.76
Completeness to max 2-theta	0.993	0.997	0.994
No. of reflections measured	65290	99797	80344
No. of independent reflections	8068	8013	8267
R(int)	0.1390	0.0648	0.0484
No. parameters / restraints	477 / 242	476 / 242	481 / 240
Final R1 values (I > 2[illegible](I))	0.0653	0.0322	0.0365
Final wR(F^2) values (all data)	0.1990	0.0949	0.1113
Goodness-of-fit on F^2	1.019	1.027	1.039
Largest difference peak & hole / e Å^{-3}	0.919, -1.161	0.502, -0.427	0.557, -0.553

* SQUEEZE has been applied to model diffuse solvent in channels along the *a* axis. SQUEEZE corrects for 186–216 electrons per unit cell, corresponding to approx. 8–10 MeCN molecules.

Table S5. Crystal and refinement data for **Ln_2** (Ln = Eu, Ho, Yb)

	**Eu_2**	**Ho_2**	**Yb_2**
CCDC number	2430475	2430472	2430478
Cambridge data number	DW_B1_0609	DW_B1_0612	DW_B2_0420
Chemical formula	$C_{56}H_{58}Eu_2N_4O_{16}$	$C_{56}H_{58}Ho_2N_4O_{16}$	$C_{52}H_{52}N_6O_{12}Yb_2$
Formula weight	1346.98	1372.92	1299.07
Temperature / K	180(2)	180(2)	180(2)
Crystal system	monoclinic	monoclinic	monoclinic
Space group	$P\ 2_1/c$	$P\ 2_1/c$	C 2/c
a / Å	9.7817(3)	9.7538(5)	23.2127(10)
b / Å	10.9697(3)	10.9893(6)	11.4677(5)
c / Å	25.8771(7)	25.7724(14)	20.0390(9)
alpha / °	90	90	90
beta / °	94.7794(12)	94.869(2)	108.591(2)
gamma / °	90	90	90
Unit-cell volume / Å^3	2767.02(14)	2752.5(3)	5056.0(4)
Z	2	2	4
Calc. density / g cm^{-3}	1.617	1.657	1.707
F(000)	1352	1368	2568
Radiation type	Cu Kα	Cu Kα	Cu Kα
Absorption coefficient / mm^{-1}	16.670	5.815	7.231
Crystal size / mm^3	0.18 x 0.10 x 0.06	0.20 x 0.10 x 0.10	0.14 x 0.10 x 0.02
2-Theta range / °	6.86-136.87	11.88-133.56	8.04-133.21
Completeness to max 2-theta	0.997	0.995	0.996
No. of reflections measured	44530	43899	46795
No. of independent reflections	5067	4868	4460
R(int)	0.0584	0.0440	0.0358
No. parameters / restraints	357 / 0	360 / 0	346 / 18
Final R1 values (I > 2[illegible](I))	0.0241	0.0248	0.0179
Final wR(F^2) values (all data)	0.0550	0.0585	0.0436
Goodness-of-fit on F^2	1.081	1.111	1.105
Largest difference peak & hole / e Å^{-3}	0.292, -0.629	0.381, -0.324	0.324, -0.624

3. Luminescence data

3.1 Excitation spectra

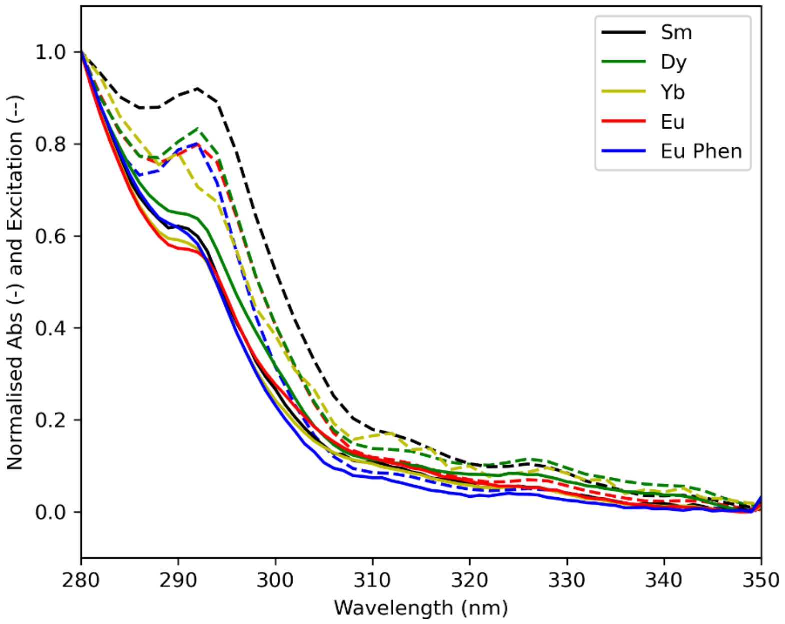

Figure S6. Comparison between the normalized absorption and excitation spectra (solution) for a selected range of compounds (**Sm_2Ti_4**, **Eu_2Ti_4**, **Dy_2Ti_4**, **Yb_2Ti_4** and **Eu_2** ['Eu Phen']), showing the overlap of ligand absorption and excitation of the lanthanide emission. The excitation spectra were recorded for the following emission wavelengths: 615 nm (Sm, Eu), 575 nm (Dy) and 980 nm (Yb).

3.2 Sample decomposition

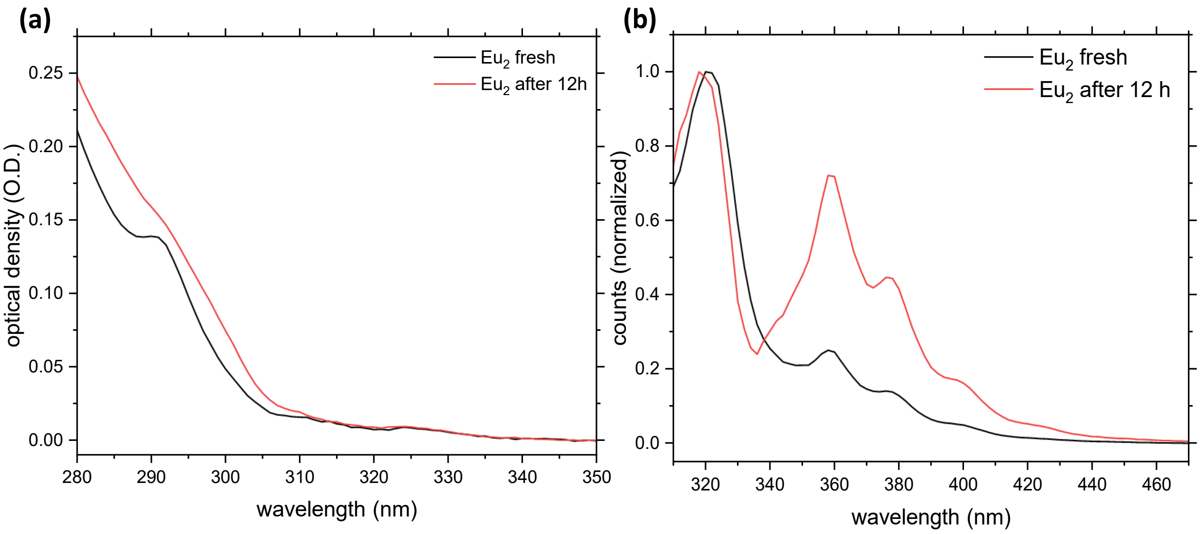

Figure S7. (a) The solution-state absorption spectrum of **Eu_2** straight after preparation and after 12 hours, showing the loss of the shoulder around 290 nm, which indicates sample degradation. The same spectral changes are observed for the **Ln_2Ti_4**-type compounds after a few days. (b) The normalized solution-state emission spectrum of **Eu_2** straight after preparation and after 12 hours showing a significant increase of the acene-like vibronic emission between 340 and 420 nm, which could suggest the dissociation of the ligand from the lanthanide over time.

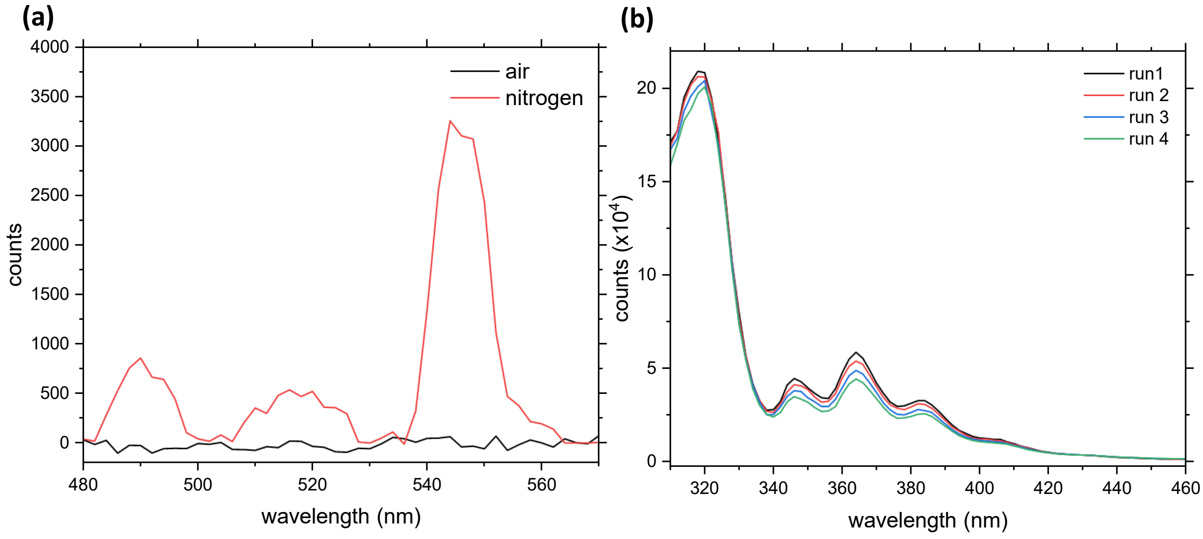

Figure S8. (a) Room-temperature emission spectrum of **Gd_2Ti_4** under air and nitrogen, showing some weak triplet emission in the absence of oxygen. The lowest-wavelength peak at 490 nm is assumed to be the 0-0 transition, which corresponds to an approximate energy of 20,408 cm^{-1} for the T_1-state of phenanthroline in the **Ln_2Ti_4**-type compounds. (b) The normalized solution-state emission spectrum of **Gd_2Ti_4** repeated after 5 (run 2), 10 (run 3) and 30 min (run 4), showing a gradual reduction of the intensity of the phenanthroline emission over time.

3.3 Residual ligand emission

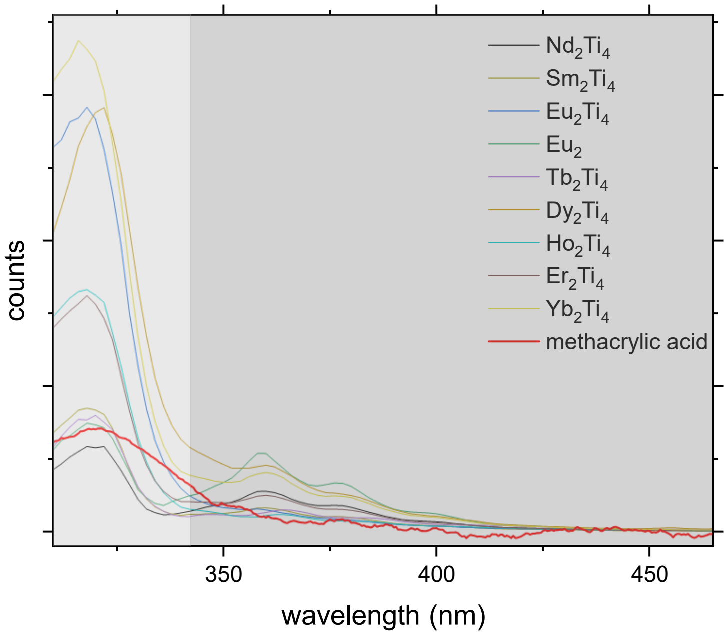

Figure S9. The solution-state (DCM) emission spectrum of the **Ln_2Ti_4**-type compounds and **Eu_2** upon excitation at 290 nm in the region of the residual ligand emission. Two distinct parts of the emission are highlighted: the typical acene vibronic emission (345-420 nm, dark grey) and a broad emission peak (310-345 nm, light grey) which matches the emission spectrum of methacrylic acid.

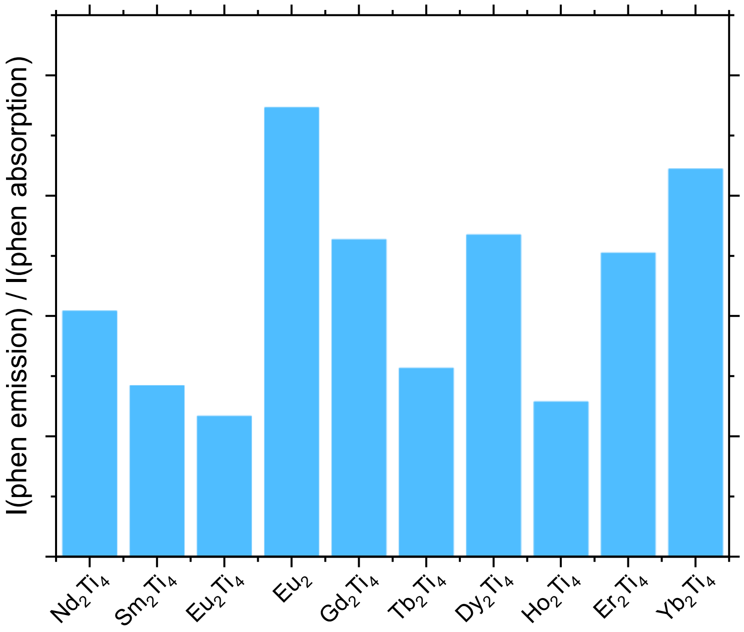

Figure S10. The intensities of the residual ligand emission I_{phen_em} in **Eu$_2$** and the **Ln$_2$Ti$_4$** cages, normalized by their respective absorption. The samples were excited at 290 nm with a 13 nm bandwidth and diluted to approx. 0.02 mg/mL (optical density < 0.3) to minimize inner and outer filter effects to make the measurements as quantitative as possible. The spectra were integrated in the following ranges: 283.5-296.5 nm (absorption) and 345-450 nm (emission).

The residual phenanthroline emission in **Gd$_2$Ti$_4$** should result from the $S_1 \rightarrow S_0$ transition only, with ISC to the T_1 state being the main quenching pathway. If I_{phen_em} is lower than for Ln=Gd, additional quenching pathways are assumed to be accessible (ET to the lanthanide, increased k_{ISC}, energy back-transfer, etc.). However, the contribution of each of the possible singlet quenching pathways could not be further identified using these data.

3.4 Lifetime measurements

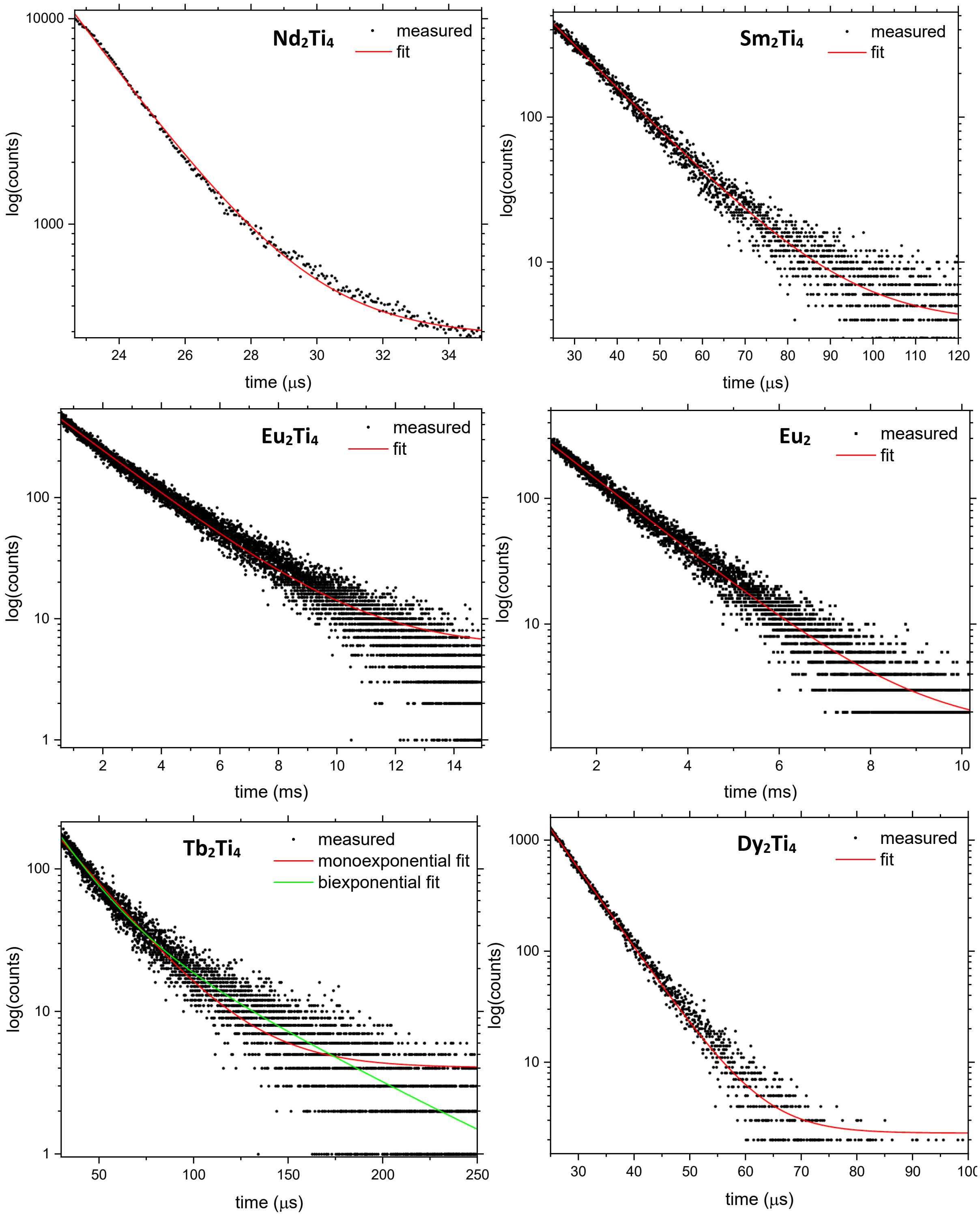

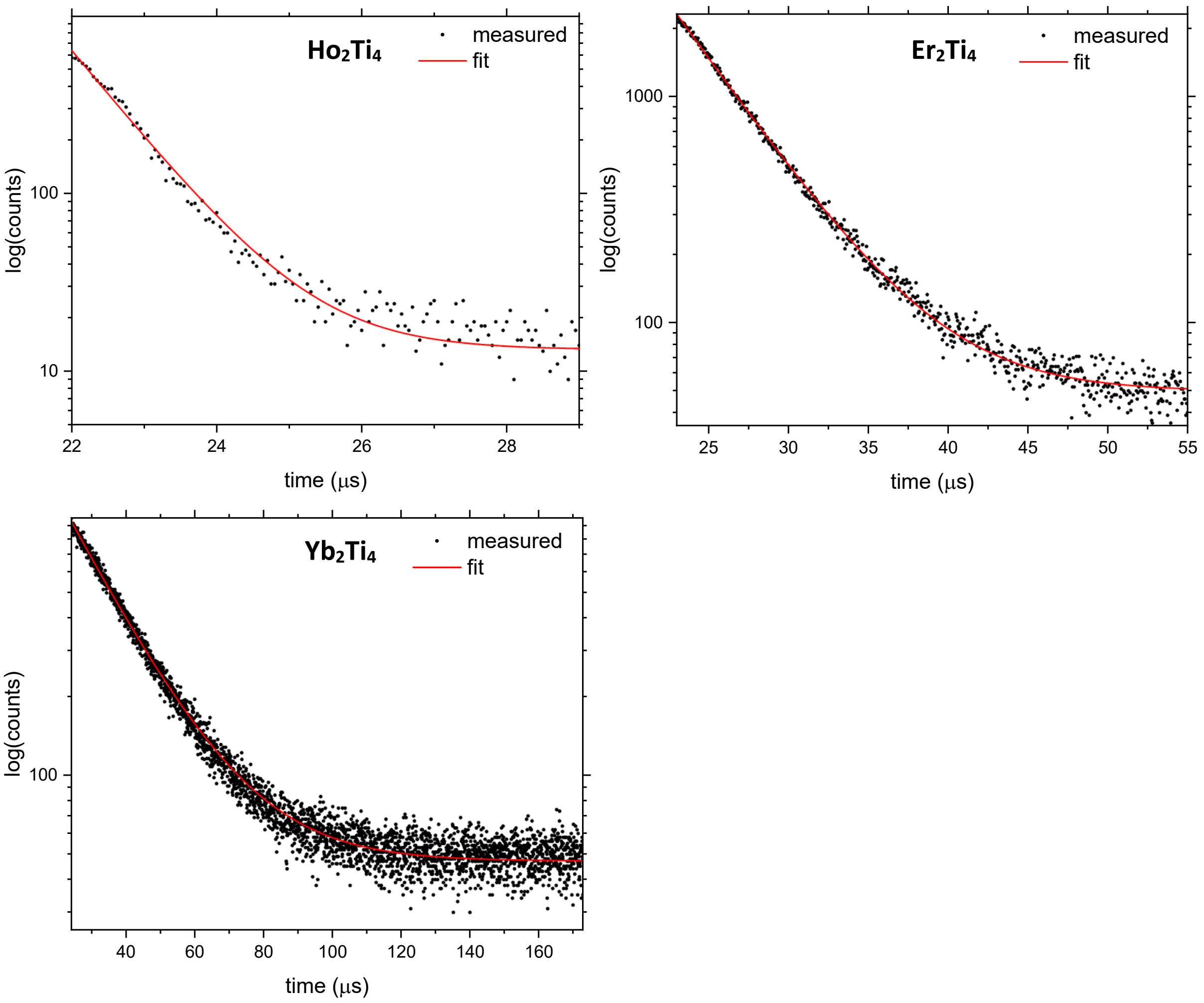

Figure S11. Exponential fits of the radiative decay for all $\mathbf{Ln_2Ti_4}$-type compounds and $\mathbf{Eu_2}$.

Table S6. The parameters of the exponential fits of the radiative decay curves of the novel $\mathbf{Ln_2Ti_4}$ compounds (Ln ≠ Eu) from which the emission lifetimes were obtained.

	X^2	B_1	τ_1 [ns]	B_2	τ_2 [ns]
$\mathbf{Nd_2Ti_4}$	1.06	7692 ± 27	1681 ± 9	755 ± 19	7523 ± 100
$\mathbf{Sm_2Ti_4}$	0.69	427 ± 2	13763 ± 76	9 ± 1	76239 ± 3735
$\mathbf{Tb_2Ti_4}$	0.78	137 ± 3	18495 ± 505	68 ± 4	50053 ± 1048
$\mathbf{Dy_2Ti_4}$	0.22	1437 ± 5	6319 ± 15	-	-
$\mathbf{Ho_2Ti_4}$	0.13	677 ± 11	757 ± 13	34 ± 2	6246 ± 176
$\mathbf{Er_2Ti_4}$	1.09	2130 ± 8	4507 ± 13	-	-
$\mathbf{Yb_2Ti_4}$	1.01	919 ± 3	16739 ± 45	-	-

Table S7. The parameters of the exponential fits of the different radiative decay curves for $\mathbf{Eu_2Ti_4}$ and $\mathbf{Eu_2}$ showing a significant increase of the lifetime for $\mathbf{Eu_2Ti_4}$.

	25 Hz decay			40 Hz decay		
	X^2	B_1	τ_1 [µs]	X^2	B_1	τ_1 [µs]
$\mathbf{Eu_2Ti_4}$	0.79	767 ± 2	2679 ± 5	1.14	416 ± 1	2626 ± 5
$\mathbf{Eu_2}$	0.53	1273 ± 3	1571 ± 3	0.63	377 ± 1	1530 ± 3

3.5 Transient absorption (TA) spectra

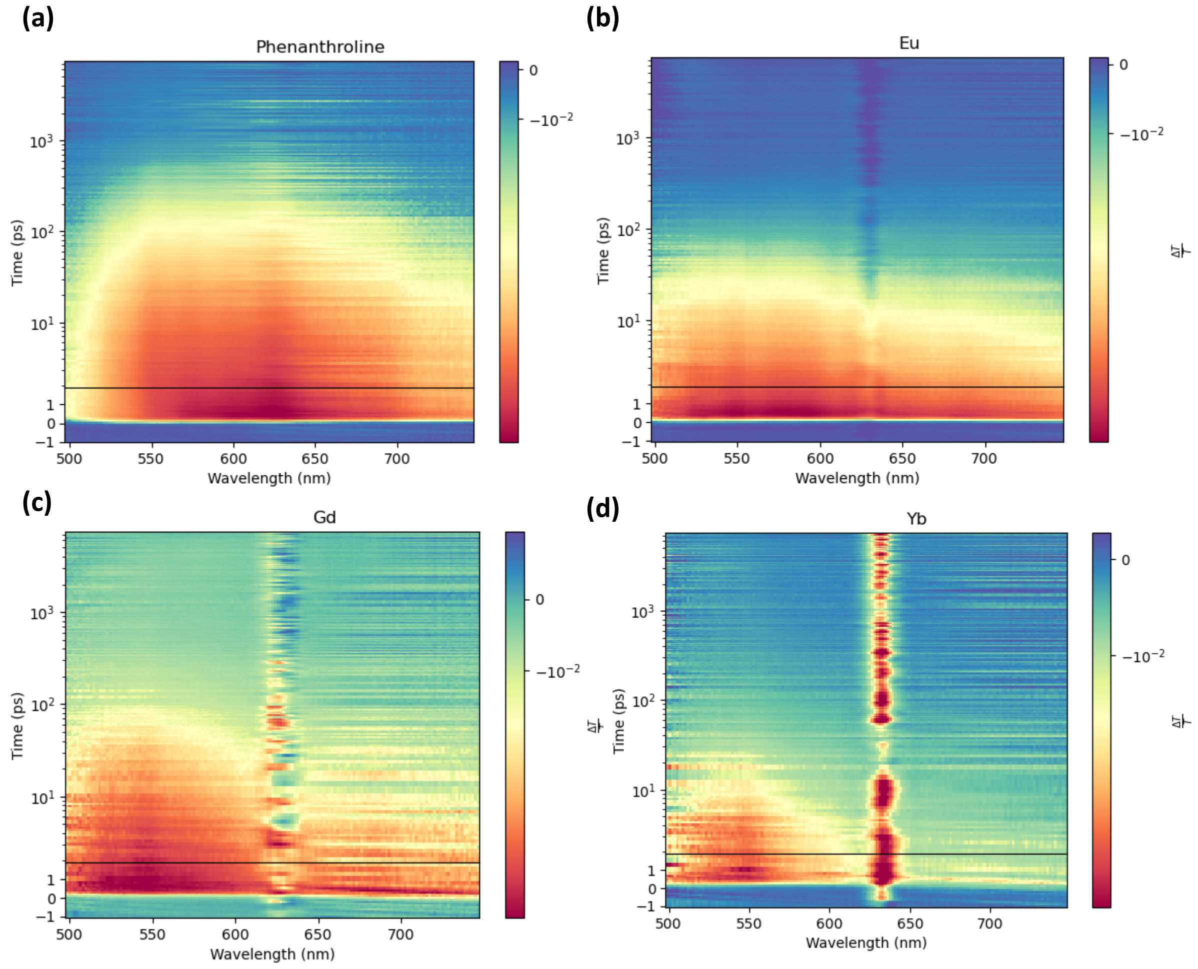

Figure S12. TA spectra of (a) free phenanthroline, (b) **Eu_2Ti_4**, (c) **Gd_2Ti_4** and (d) **Yb_2Ti_4** (pumping at 320 nm, sample concentration around 1 mg/mL). For the free phenanthroline even a highly concentrated sample (30 mM) only gave one weak signal around 620 nm with no sign of a triplet state growing in, which indicates that the ligand itself it difficult to analyze in a pump-probe experiment. For the three **Ln_2Ti_4**-type compounds similar spectra were obtained with one signal appearing in the detectable probing range around 550 nm, which peaks at 500 ps and fully decays after 7.5 ns.

Table S8. Lifetimes of the excited state of the coordinated phenanthroline in the **Ln_2Ti_4**-type compounds and as a free ligand obtained from TA measurements.

	**phenanthroline**	**Eu_2Ti_4**	**Gd_2Ti_4**	**Yb_2Ti_4**
lifetime [ns]	1.62	0.15	2.14	1.74
$\Phi_{ligand \rightarrow Ln}$ [%]	-	93	-	19

Looking at the lifetimes of the ligand excited state the **Eu_2Ti_4** compound shows the shortest lifetime which suggests that the absorbed energy is quickly passed onto the Eu^{3+} centre, allowing efficient sensitization. **Yb_2Ti_4** has a lifetime similar to that of free phenanthroline which is consistent with the small amount of energy transfer suggested by the low intensity of the lanthanide emission. The fact that the lifetime in **Gd_2Ti_4** is longer than that of the free ligand, however, is surprising. In general, upon coordination to a highly paramagnetic ion such as Gd^{3+}, the rate of ISC in a molecule increases.[3] This

should decrease the excited state lifetime of phenanthroline in **Gd_2Ti_4**, whether the state is a singlet or a triplet. Given this does not appear to be the case here, we suggest that coordinating to Gd^{3+}, in which the excited states lie too high in energy to interact with those of phenanthroline, reduces non-radiative deactivation of the ligand excited state, which leads to the increase of its lifetime.

3.5 Aggregation studies

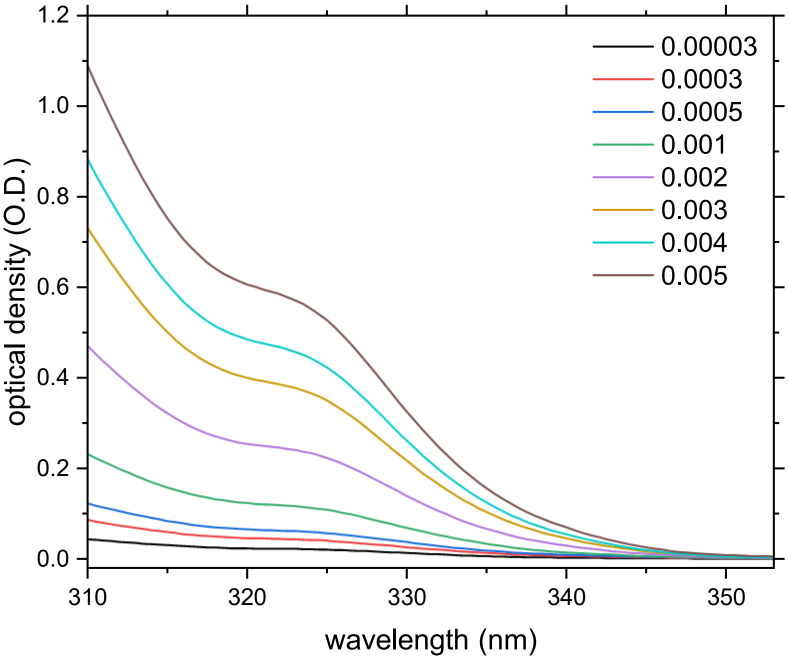

Figure S13. Solution-state UV-Vis absorption spectra of phenanthroline at varying concentrations (mol/L in DCM), showing the formation of a shoulder between 320 and 330 nm at higher concentrations. This change could indicate the formation of aggregates. Since the TA measurements shown above were carried out at 0.03 mol/L an even higher degree of aggregation can be assumed for these samples, which potentially contributed to the complicated detection of any excited states in the molecule.

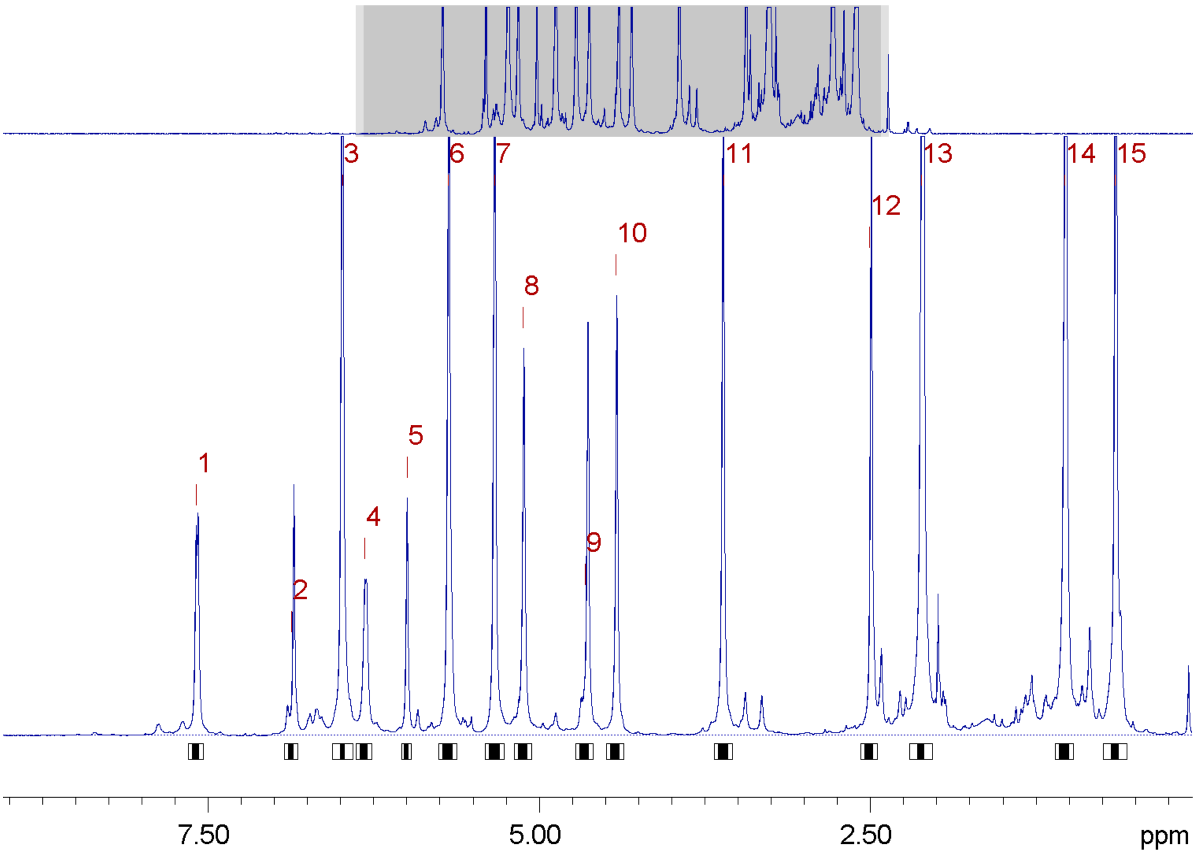

Figure S14. The ^{1}H-NMR spectrum of **Eu_2Ti_4** in CD_2Cl_2 used for a DOSY (Diffusion-Ordered Spectroscopy) experiment to determine the diffusion coefficient of the molecule in solution.

Table S9. Summary of the fitting parameters for each peak.

Peak name	F2 [ppm]	Io	error	D [m2/s]	error
1	7.591	2.13e+09	2.558e+05	7.06e-10	1.931e-13
2	6.872	1.50e+09	2.395e+05	6.87e-10	2.511e-13
3	6.484	8.17e+09	2.941e+05	6.98e-10	5.723e-14
4	6.322	1.98e+09	2.576e+05	7.24e-10	2.136e-13
5	6.001	1.29e+09	1.995e+05	7.00e-10	2.468e-13
6	5.690	5.84e+09	2.764e+05	7.07e-10	7.615e-14
7	5.337	8.19e+09	4.480e+05	3.29e-09	3.687e-13
8	5.124	2.90e+09	2.717e+05	7.27e-10	1.545e-13
9	4.657	2.88e+09	2.701e+05	7.09e-10	1.512e-13
10	4.424	2.86e+09	2.698e+05	7.06e-10	1.515e-13
11	3.612	4.48e+09	2.757e+05	7.01e-10	9.818e-14
12	2.511	4.02e+09	2.628e+05	7.07e-10	1.053e-13
13	2.116	1.78e+10	3.129e+05	7.05e-10	2.823e-14
14	1.034	1.12e+10	2.769e+05	7.11e-10	3.992e-14
15	0.652	9.53e+09	3.120e+05	6.97e-10	5.206e-14

From the data shown above an average diffusion coefficient of D = 7.06×10^{-10} m^2/s was obtained (excluding peak 7). Using the Stokes-Einstein-Sutherland equation the hydrodynamic radius r was calculated as follows:

$$r = \frac{k_B T}{6\pi\eta D}$$

With k_B = Boltzmann constant, T = 298 K and $\eta(CD_2Cl_2)$ = 0.000432 kgm/s, this expression gives:

$$r = 7.31 \text{ Å}$$

The single-crystal structure of **Eu_2Ti_4** suggests a diameter of around 13 Å (methacrylate – methacrylate) or 17 Å (phenanthroline – phenanthroline), which makes this value a reasonable fit for a single molecular compound. This suggests that no aggregation takes place in the solutions of the **Ln_2Ti_4**-type compounds in the concentration range used for the optical measurements shown above.

References

[1] X.-P. Shu, W. Luo, H.-Y. Wang, M.-Y. Fu, Q.-Y. Zhu, J. Dai, *Inorg.Chem.* **2020**, *59*, 10422.

[2] A. L. Spek, *Acta Cryst.* **2015**, *C71*, 9-18.

[3] P. Yuster, S. I. Weissman, *J. Chem. Phys*. **1949**, *17*, 1182.
